# Biochemical Studies of a Cyanobacterial Halogenase Support the Involvement of a Dimetal Cofactor

**DOI:** 10.1021/acs.biochem.4c00720

**Published:** 2025-04-29

**Authors:** Michelle L. Wang, Nathaniel R. Glasser, Mrutyunjay A. Nair, Carsten Krebs, J. Martin Bollinger, Emily P. Balskus

**Affiliations:** † Department of Chemistry and Chemical Biology, 1812 Harvard University, Cambridge, Massachusetts 02138, United States; ‡ Department of Chemistry, 311285 The Pennsylvania State University, University Park, Pennsylvania 16802, United States; § Department of Biochemistry and Molecular Biology, The Pennsylvania State University, University Park, Pennsylvania 16802, United States; ∥ Howard Hughes Medical Institute, Harvard University, Cambridge, Massachusetts 02138, United States

## Abstract

Halogenation is a prominent transformation in natural product biosynthesis, with over 5000 halogenated natural products known to date. Biosynthetic pathways accomplish the synthetic challenge of selective halogenation, especially at unactivated *sp* ^3^ carbon centers, using halogenase enzymes. The halogenase CylC, discovered as part of the cylindrocyclophane (*cyl*) biosynthetic pathway, performs a highly selective chlorination reaction on an unactivated *sp* ^3^ carbon center and is proposed to use a dimetal cofactor. Putative dimetal halogenases are widely distributed across cyanobacterial biosynthetic pathways. However, rigorous *in vitro* biochemical and structural characterization of these enzymes has been challenging. Here, we report additional bioinformatic analyses of putative dimetal halogenases and the biochemical characterization of a newly identified CylC homologue. Site-directed mutagenesis identifies highly conserved putative metal-binding residues, and Mössbauer spectroscopy provides direct evidence for the presence of a diiron cofactor in these halogenases. These insights suggest mechanistic parallels between diiron and mononuclear nonheme iron halogenases, with the potential to guide further characterization and engineering of this unique subfamily of metalloenzymes.

## Graphical abstract

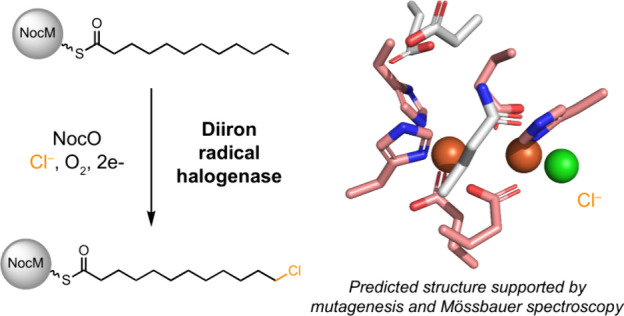

Microbial natural product biosynthesis uses a broad range of enzymatic transformations to generate structurally complex, bioactive products.^1^ One of the most striking and versatile microbial biosynthetic transformations is halogenation. Halogenation is widespread across all domains of life, with over 5000 halogenated natural products identified.^2^ This modification alters the physiochemical properties and bioactivity of molecules, with approximately 25% of all drugs and drug candidates containing a C–X (where X = F, Cl, Br, or I) bond.^3^ While synthetic chemists face significant challenges in selective halogenation,^4^ Nature has evolved halogenases, enzymes that perform this chemistry with remarkable efficiency and selectivity.

All well-characterized halogenases belong to one of three major classes: electrophilic (C–X bond formed by nucleophilic attack on an electrophilic RO–X species), radical nonheme mononuclear iron (C–X bond formed by selective radical transfer), and SAM-dependent nucleophilic (C–X bond formed by S_N_2 substitution).^5^ There has been significant interest in the characterization and discovery of enzymes that halogenate unactivated C­(*sp* ^3^)–H bonds. For many years this type of reactivity was thought to be unique to nonheme mononuclear iron halogenases, which utilize oxygen, α-ketoglutarate, and a mononuclear iron cofactor to form the C–X bond (where X = Cl or Br) (Figure 1A). SyrB2, reported by Walsh and co-workers in 2005,^6^ was the first example of enzymatic oxidative halogenation in nonheme iron enzymes, enabling the discovery of many related enzymes.^7–9^ The crystal structure of SyrB2, solved by Drennan and Walsh, contained a 2 His–Cl facial triad, with the chloride replacing the carboxylate ligand of the Asp residue found in analogous hydroxylases.^10^ This structural insight highlighted the possibility of a mechanism involving the iron-bound chloride. Indeed, subsequent mechanistic studies of these halogenases determined that reactive ferryl–oxo intermediates selectively abstract hydrogen atoms from unactivated C­(*sp* ^3^) centers and C–X bonds are formed by a highly selective radical rebound with the halide ligated to the iron center.^7,10–12^ These fundamental studies have informed engineering of radical halogenases, expanding substrate scope to include non-natural, free-standing substrates and altering reactivity to enable bromination and azidation.^13,14^

**Figure 1:**
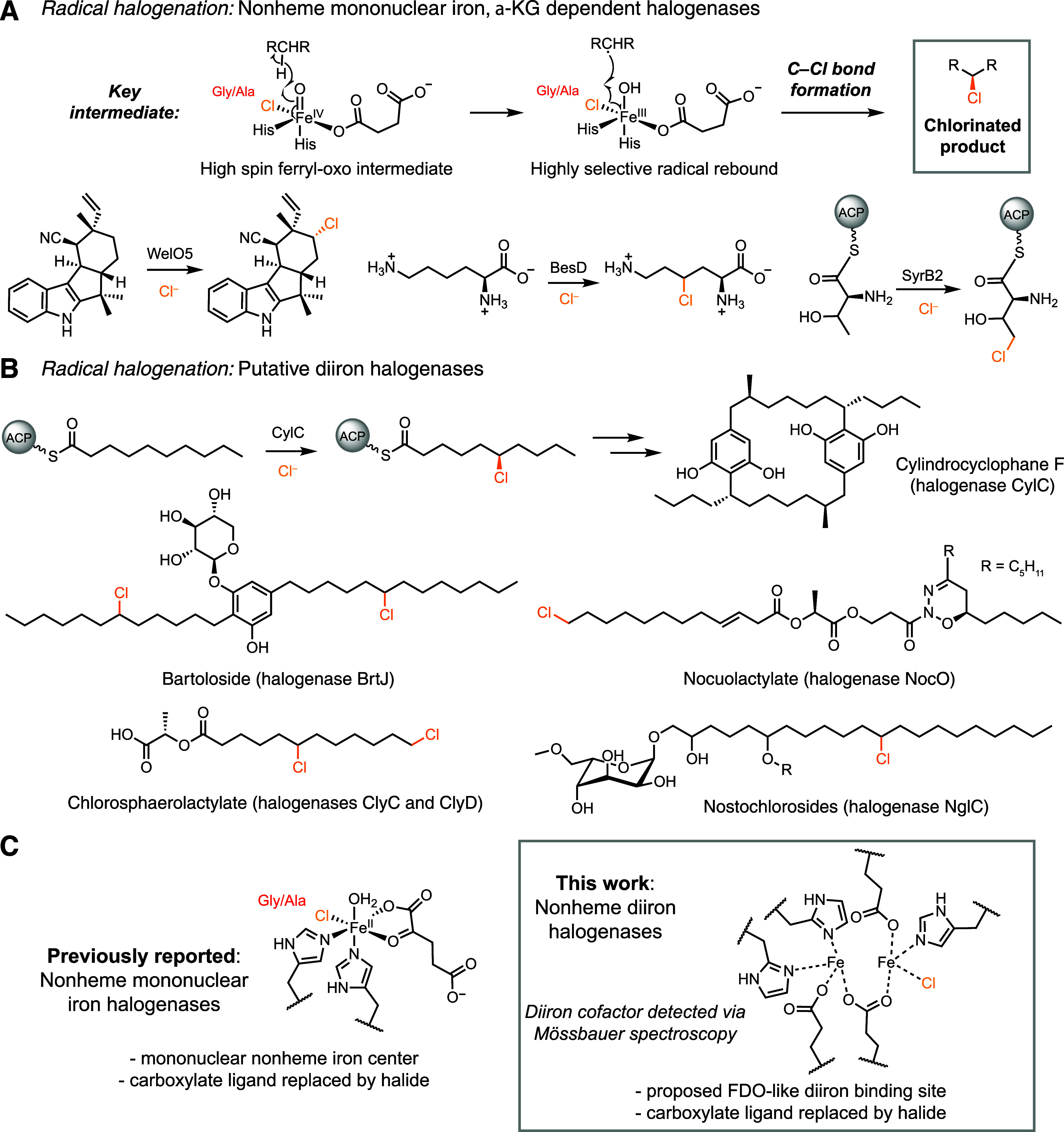
Radical halogenases functionalize unactivated carbon centers. (A) Overall reaction and examples of radical halogenation by mononuclear nonheme iron halogenases. (B) Putative diiron halogenases biosynthesize fatty acid-derived cyanobacterial natural products. (C) Characterization of active site residues and spectroscopy suggest putative diiron halogenases use a diiron cofactor for radical halogenation.

In 2017, we discovered a new radical halogenating enzyme in the biosynthesis of cylindrocyclophane F by the Cyanobacterium Cylindrospermum lichenforme (Figure 1B).^15^ Encoded within the *cyl* biosynthetic gene cluster (BGC), CylC performs a highly regio- and stereoselective chlorination at C_6_ of its acyl carrier protein (ACP)-tethered substrate, CylB-decanoyl thioester. The resulting *R*-configured alkyl chloride is further elaborated to a chlorinated alkyl resorcinol intermediate, which undergoes a C–C bond forming dimerization by the Friedel–Crafts alkylating enzyme CylK to form the paracyclophane scaffold.^15,16^ Initial bioinformatic analyses revealed that CylC does not share any amino acid sequence similarity with known radical halogenases. While its halogenation activity was iron-dependent, it did not require cofactors or cosubstrates associated with enzymatic halogenation (heme, flavin, vanadate, α-ketoglutarate, *S*-adenosyl methionine). Instead, CylC resembles the ferritin-like diiron oxygenase AurF, which performs a 6-electron *N*-oxidation of *p*-aminobenzoic acid to *p*-nitrobenzoic acid in aureothin biosynthesis.^17^ Despite low amino acid similarity (26%) and identity (12%) between the two enzymes, aligning an initial homology model of CylC with AurF’s crystal structure identified a set of putative CylC metal binding residues in the location of AurF’s diiron metallocofactor, leading to the hypothesis that CylC also possesses a dimetal cofactor. Initial biochemical experiments provided support for the hypothesis that CylC is a diiron enzyme by revealing an iron:enzyme ratio of ∼2:1.^15^

Ferritin-like diiron oxygenases and oxidases (FDOs) are nonheme diiron enzymes that perform a wide variety of chemical reactions by activating molecular oxygen, inducing a transition from a typical diferrous resting state to a diferric state.^18,19^ Conversion of the corresponding diiron–O_2_ adduct to various reactive intermediates enables oxidative transformations including *N*-oxygenation,^17^ alkane hydroxylation,^20^ site-selective desaturation,^21^ and alkene epoxidation.^22^ Despite this broad reaction scope, diiron enzymes are not known to perform halogenation, potentially making CylC the founding member of a new FDO subfamily.

Subsequent studies of putative diiron halogenases have largely focused on their genomic distribution and their associations with natural products. A survey of genomes and a PCR screen of cyanobacterial isolates revealed that CylC homologues are widespread across all major cyanobacterial classes, making them a major C–H functionalization strategy in the phylum. They are encoded within BGCs that are predicted to give rise to diverse molecular architectures^23^ and participate in the biosyntheses of additional chlorinated fatty acid-derived natural products and biosynthetic intermediates (Figure 1B).^24–28^

Despite the prevalence of putative diiron halogenases, little is known about the biochemical and structural basis for their halogenation activity. Beyond the previous preliminary studies of CylC, no reports have characterized the metallocofactor or key catalytic or metal binding residues of these enzymes. Notably, *in vitro* biochemical and structural experiments using these enzymes have been hindered by their general intractability and insolubility: initial studies of CylC required two coexpression partners, the fatty acid activating ligase (FAAL) CylA and the ACP CylB, to obtain low levels of soluble enzyme from anEscherichia coli expression system. Given that putative diiron halogenases represent a major C–H functionalization strategy in cyanobacteria, gaining a more detailed understanding of their structures and mechanisms would provide valuable insights into a chemically important biosynthetic transformation and new mode of reactivity for the nonheme diiron cofactor.

Here, we use bioinformatic, biochemical, and spectroscopic approaches to identify a proposed mode of diiron binding among dimetal halogenases. Multiple sequence alignments and structural predictions of CylC homologues reveal a highly conserved putative diiron binding site that may contain an open site for chloride binding. Using site-directed mutagenesis of the CylC homologue NocO, we show these proposed active site residues likely contribute to metal binding and chlorination activity. The lack of activity in these variants suggests the relevance of these residues in metallocofactor assembly. Finally, we use Mössbauer spectroscopy to obtain direct evidence of a diiron cofactor in NocO. Together, this work provides experimental support for the involvement of a diiron metallocofactor in this subfamily of radical halogenases (Figure 1C).

To guide further biochemical studies of putative diiron halogenases, we aligned the amino acid sequences of CylC, selected homologues, and the diiron *N-*oxygenase AurF (Figure 2A, Figure S1). This analysis identified candidate active site residues in the diiron halogenases based on the previously established iron binding ligands in AurF.^29^ We located nine residues, seven of which are found in a QExxH and HxxDExxH motif, that most likely comprise the active site in the selected homologues. A further examination of 241 other CylC homologues identified in a BLAST search revealed that these nine residues are conserved in the dimetal halogenase subfamily (Figure 2A, Figure S2). We used both AlphaFold2 and AlphaFold3 to generate predicted structures of CylC and superimposed them onto the crystal structure of diiron-bound AurF to identify roles for these residues. Consistent with the prior homology model, CylC is predicted to have the distinctive four α-helix bundle characteristic of FDOs, with a glutamate- and histidine-rich active site (Figure 2B, Figures S3, S4).^18,30^ The AlphaFold structures predicted roles in metal binding and hydrogen bonding for the nine residues we had identified in our sequence alignment. In AurF’s crystal structure, two aspartates (D135 and D226) act as bases and hydrogen bond acceptors for H230 and H139, respectively, allowing the two histidine residues to be stronger ligands for the diiron cofactor.^29^ In CylC and its homologues, D135 is replaced by a glutamine, which lacks the basicity of an aspartate residue, potentially altering the metal binding environment in dimetal halogenases. However, there are differences in the predicted metal binding ligands. The 3 histidine/4 carboxylate diiron binding mode of AurF is largely unique to the *N-*oxygenase subfamily of FDOs, with most other FDOs having a six-coordinate iron-binding environment. Specifically, most nonheme diiron hydroxylases bind the diiron cofactor using a 2 histidine/4 carboxylate motif.^31^ The AlphaFold structure predicts that CylC has six primary metal binding ligands as compared to AurF’s seven. However, in contrast to other C–H oxidizing FDOs, the metal binding environment of CylC is predicted to contain a 3 histidine/3 glutamate motif resembling AurF’s active site. Notably, the final coordination site in the predicted structures of CylC and other homologues, occupied by E196 in AurF and E283 in the original CylC homology model, is predicted to be left unoccupied by a shift of this glutamate to outside the primary metal binding sphere (Figures S4, S7). Docking two iron atoms and a chloride ion with AlphaFold3 suggested that this predicted open coordination site could accommodate a chloride ion, a structural feature reminiscent of the chloride binding site of nonheme mononuclear iron halogenases (Figure 2B).^10^ Inspecting the sequence alignment of CylC homologues revealed that this glutamate is the only residue among the nine identified that is not absolutely conserved; a small subset of sequences have glutamine at this position (Figure S2). A search of residues within 5 Å of the putative chloride-bound iron atom did not reveal any additional residues that could act as metal-binding ligands, further supporting the possibility of halide coordination at this site (Figures S7, S8). By predicting features distinct from AurF and the original CylC homology model, these analyses potentially redefine the active sites of putative dimetal halogenases.

**Figure 2:**
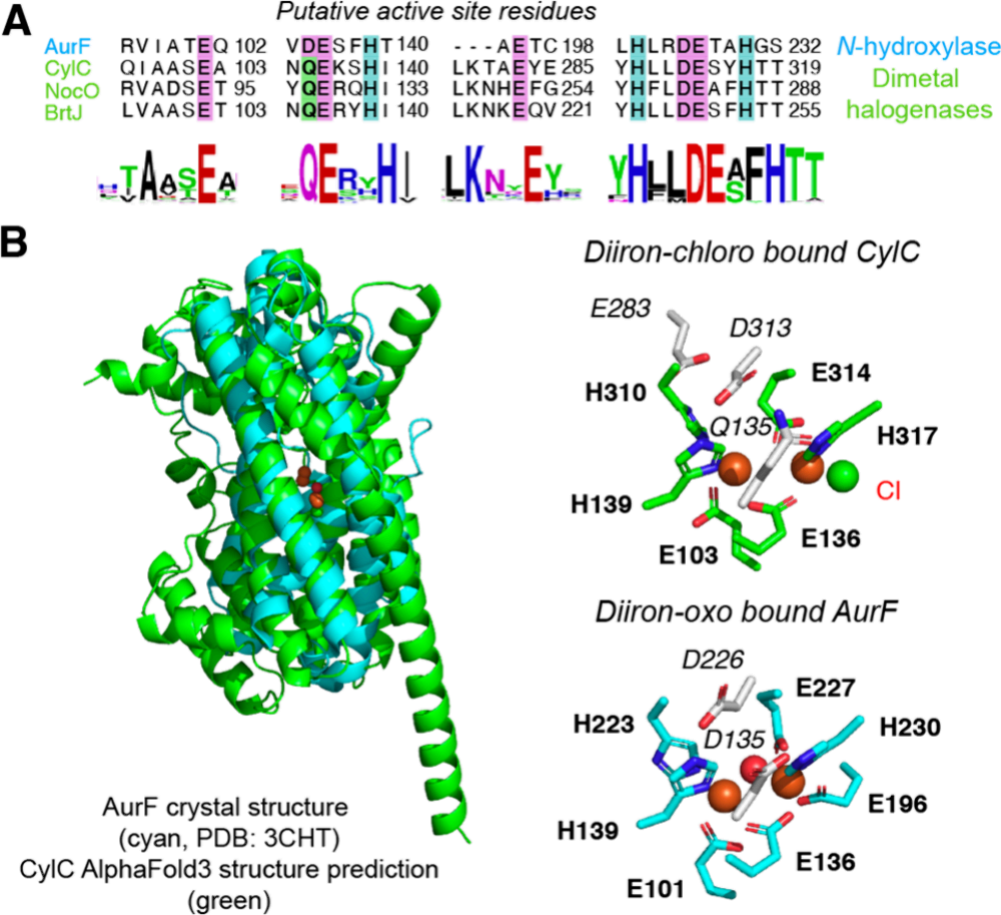
Bioinformatic analyses of CylC homologues predict amino acids involved in dimetal cofactor binding and identify a potential chloride binding site. (A) Protein sequence alignment of AurF with select CylC homologues reveals a set of putative active site residues. (B) AlphaFold3 predicted structure of CylC contains the FDO-like fold found in AurF and a dimetallocofactor with an open coordination site for halide binding. Italicized residues represent active site residues that are predicted to not directly participate in metal binding.

To functionally characterize the putative active site residues in CylC homologues, we sought a more tractable enzyme for *in vitro* studies. As highlighted above, biochemical studies of CylC have been complicated by its poor solubility, instability, and strict need for two coexpression partners.^15^ Therefore, we screened 12 phylogenetically diverse CylC homologues^23^ for improved solubility and expression in E. coli (Table S3). We attempted heterologous expression under a variety of conditions, including different host strains, temperatures, promoters, and coexpression with a cognate ACP. This screening effort identified one homologue, NocO (43% amino acid ID, 60% similarity), fromNodulariasp. LEGE 06071, with improved expression characteristics, namely its ability to be solubly expressed with just its cognate ACP, NocM (Figure 3A). We selected NocO as a model system to study dimetal halogenases.

**Figure 3:**
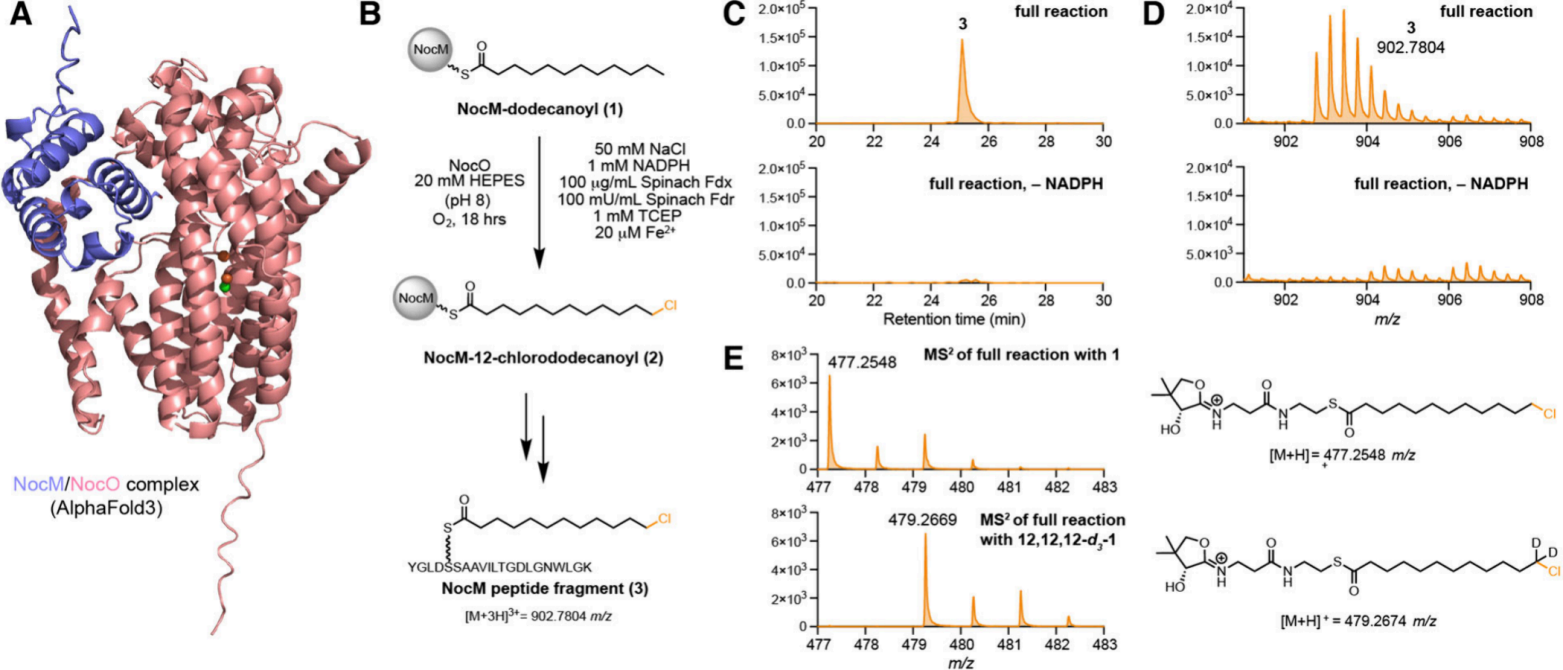
NocO chlorinates the terminal position of NocM-tethered dodecanoyl thioester. (A) AlphaFold3 predicted structure of the NocM/NocO complex. (B) *In vitro* assay for halogenation of **1** by NocO (Fdx = ferredoxin, Fdr = ferredoxin reductase). (C) Representative extracted ion chromatogram (EIC) of trypsin digested product **3** in full *in vitro* reactions and reactions lacking the electron source NADPH. (D) Representative MS spectra of **3** extracted from EICs in (C). (E) MS/MS fragmentation of product in reactions supplemented with NocL-loaded NocM-12,12,12-*d*_3_-dodecanoyl thioester shows NocO selectively halogenates the terminal carbon of the acyl chain of **1**. Assays and negative controls were performed in triplicate for experiments in (C) and (D), assays and negative controls were performed in duplicate for the isotopic labeling experiment in (E).

NocO is encoded by the *noc* BGC which produces the nocuolactylates.^27^ It is hypothesized to chlorinate an ACP-tethered dodecanoyl thioester starter unit (**1)** in the assembly of the chlorosphaerolactylate half of the nocuolactylates.^26,27^ We expressed and purified NocO together with its ACP, NocM, as *N*-terminally His_6_-tagged constructs from E. coli BL21­(DE3) (Figure S9). Since NocM was purified in its apo form, we used the phosphopantetheinyl transferase Sfp to generate **1** from dodecanoyl CoA (Figure 3B). We reconstituted NocO’s chlorination activity toward **1** *in vitro* by combining the enzyme–substrate mixture with redox components required for CylC activity.^16^ Reactions supplemented with an exogenous ferrous source (Figure 3C, 3D) and reactions with the as-purified protein (Figure S14) all generated monochlorinated **2**. When a terminally deuterated version of **1** was tested, a + 2 *m/*z shift was observed in the phosphopantetheine ejection fragment, confirming NocO’s selectivity for the terminal position of its substrate (Figure 3E). We were unable to detect hydroxylated products in NocO assays, a known chemoselectivity issue in nonheme mononuclear iron halogenases.^32–35^ The reconstitution of NocO’s chlorination activity confirmed its role as a halogenase.

We next set out to investigate the importance of the predicted metal binding residues in NocO. However, the previously established heterologous expression system failed to yield appreciable amounts of soluble mutant enzymes, which prevented biochemical experiments. Similar expression difficulties were reported for AurF upon mutation of its metal-binding active site residues.^36^ We made several attempts to increase protein yield, including scaling up expression cultures, expressing a SUMO-tagged construct, and coexpression with both NocM and the FAAL NocL, without success. We next explored changing the heterologous expression host and discovered that E. coli ArcticExpress (DE3), a BL21 derivative that constitutively expresses the chaperones Cpn60 and Cpn10 fromOleispira antarctica, produced our variants at comparable levels to wild-type NocO when coexpressed with NocM (Figure S17). The two proteins also copurify with Cpn60 (Figure S18). Using this expression system, we reconstituted wild-type NocO’s *in vitro* chlorination activity (Figure 4C). In addition, a Ferene-S assay revealed approximately 1.97 mol Fe/mol in wild-type NocO, which is consistent with a diiron cofactor. This observation, in combination with NocO’s homology with CylC and its demonstrated halogenation activity, supports the assignment of NocO as a dimetal halogenase.

**Figure 4:**
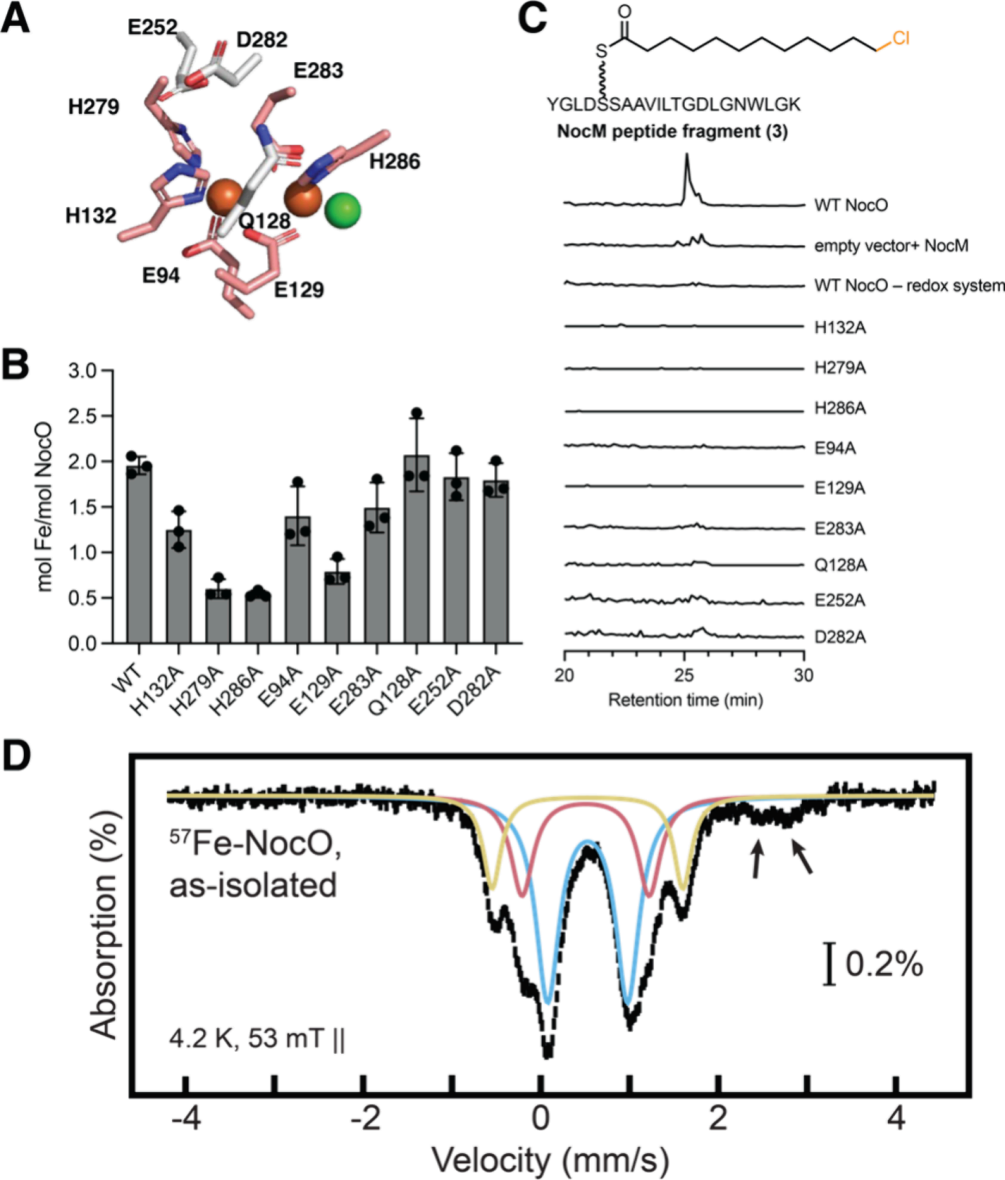
Mutation of predicted metal binding residues and Mössbauer spectroscopy support the presence of a diiron cofactor in NocO. (A) Putative active site residues in NocO identified in an AlphaFold3-predicted structure. Residues shown in white are predicted to be not directly involved in metal binding. (B) Representative EICs for **3** show that active site variants are catalytically inactive. (C) Certain active site variants show decreased iron loading relative to WT NocO. Assays and corresponding negative controls were performed in triplicate, bars represent mean ± SD. (D) 4.2-K/53-mT Mössbauer spectrum of ^57^Fe-enriched, aerobically isolated NocO. The experimental spectrum is depicted in black vertical bars of heights reflecting the standard deviations of the absorption values during spectral acquisition. The blue, red, and yellow lines are quadrupole doublet simulations illustrating the fractional contributions from the different diiron­(III)-species quoted in the text. The black arrows represent the high-energy lines of the Fe­(II)-species, as quoted in the text. The parameters of the different iron-species used for simulation are summarized in Table S7.

Substituting alanine in place of any of the nine putative active site residues revealed that all of them were essential for chlorination activity (Figure 4A,C). However, only the six variants that replace a proposed metal binding ligand showed reduced iron binding relative to wild-type, consistent with a near single occupancy-like state (Figure 4B). Interestingly, the Q128A, E252A, and D282A variants all had comparable iron stoichiometry relative to the wild-type enzyme. This finding supports the hypothesis that E252 is likely not a metal binding residue and instead suggests that D282 and E252 may act as hydrogen bond acceptors for their proximal iron-binding histidine residues and that their mutation to alanine may hinder catalytic activity without affecting metal acquisition. While these iron:protein stoichiometries are approximate due to the challenge of accurately quantifying NocO in our enzyme preparations, the absence of chlorination activity in these variants is consistent with their proposed roles as first- and second-sphere cofactor ligands.

To directly investigate the nature of NocO’s metallocofactor, we purified ^57^Fe-enriched NocO on a large scale for Mössbauer spectroscopy. The UV/vis absorption spectrum of the as isolated sample exhibited broad absorbance features at ∼ 360 nm and ∼ 410 nm (Figure S11), of which the former is likely characteristic of an oxo-to-iron ligand-to-metal charge transfer band found in other members of the FDO family, typically indicative of a μ-oxo-Fe_2_^III/III^ species.^37^ The 4.2-K/53-mT spectrum of the ^57^Fe-enriched, aerobically isolated protein (Figure 4D, black vertical bars) can be simulated as three partially resolved quadrupole doublets with isomer shifts (δ) and quadrupole splitting (Δ*E*_Q_) (δ_1_ = 0.53 mm/s, Δ*E*_Q1_ = 0.90 mm/s, 50% of total absorption, blue line; δ_2_ = 0.51 mm/s, Δ*E*_Q2_ = 1.43 mm/s, 23% of total absorption, red line; and δ_3_ = 0.53 mm/s, Δ*E*_Q3_ = 2.15 mm/s, 17% of total absorption, yellow line) characteristic of antiferromagnetically coupled high-spin diferric clusters with diamagnetic (*S* = 0) electron-spin ground states (Table S7).^38,39^ The unusually large quadrupole splitting of the species outlined in yellow suggests the presence of a μ-oxo-bridge,^40^ as has been observed in other FDOs, such as the β subunit of class Ia ribonucleotide reductase from Escherichia coli^41–43^ and the *N*-oxygenase AurF.^44^ In addition, there are two weak absorption features at ∼2.5 and ∼2.8 mm/s, positions typical for the high-energy lines of quadrupole doublets arising from high-spin Fe­(II) ions (Figure 4D, black arrows). The spectrum of an equivalent sample reduced anoxically with excess sodium dithionite (Figure S12, black vertical bars) shows an increased intensity from these absorption features, which contribute ∼ 60% of the total absorption area and have parameters (δ ∼ 1.2 mm/s, Δ*E*_Q_ ∼ 3.0 mm/s) typical of nitrogen and oxygen coordinated high-spin Fe­(II) species.^38,44,45^ In the reduced sample, two minor absorption features (with ∼20% of the total absorption area each) with their high-energy lines at positions ∼1 and ∼1.6 mm/s, (Figure S12, black arrows) show that the diferric species remain after reduction only by dithionite, suggesting the need for a mediator to access the buried cofactor. The Mössbauer data thus provide definitive evidence for the presence of a nonheme diiron cofactor in NocO.

In summary, these findings support the involvement of a diiron cofactor in a distinct group of radical halogenases. By combining information from multiple sequence alignments and predicted enzyme structures, we identified a conserved set of putative metal binding residues and a potential open coordination site for chloride binding in CylC and its homologues. In addition, the predicted 3 histidine/3 glutamate architecture is distinct from that of diiron alkane hydroxylases and *N*-oxygenases, the closest structurally characterized homologues of this halogenase family.^29,46^ The predicted change of the aspartate hydrogen bond acceptor in FDOs to a glutamine in dimetal halogenases may also have implications for chemoselectivity and mechanism. Further studies will be needed to determine the exact nature of the ligands bound to the diiron cofactor, but the conservation of these identified residues suggests that they are functionally important.

To overcome difficulties heterologously expressing CylC, we evaluated multiple homologues, ultimately identifying NocO as the most viable candidate for biochemical studies. NocO purified from E. coli catalyzes a highly chemo- and regioselective chlorination of the terminal carbon of an ACP-tethered dodecanoyl thioester. Mutagenesis of active site residues has been important for understanding mononuclear nonheme Fe-dependent halogenases,^47^ but has been challenging in FDOs.^36^ The mutagenesis of putative metal-binding residues in NocO suggests they are essential for chlorination activity and diiron metallocofactor assembly. Importantly, the Mössbauer spectrum of as-isolated NocO exhibits primarily quadrupole doublet features indicative of antiferromagnetically coupled diiron­(III) clusters, providing the first direct spectroscopic evidence for the identity of the metallocofactor in this group of halogenases.

Our results provide exciting insights into the potential mechanism and evolution of these enzymes. The high bond dissociation energies of C­(*sp* ^3^)–H bonds (∼98–101 kcal/mol)^48^ and the unactivated nature of the carbon centers functionalized by diiron halogenases necessitates a radical-based halogenation mechanism.^2^ The enzymatic halogenation of C­(*sp* ^3^)–H bonds has been of longstanding interest to both biological and synthetic chemists. From the initial characterization of the mononuclear Fe-dependent radical halogenases^11^ to recent reports of a dicopper radical halogenase,^49^ these halogenases are proposed to share certain mechanistic features. In mononuclear Fe-dependent radical halogenases, the carboxylate ligand found in related hydroxylases is mutated to a smaller, nonpolar residue to allow the binding of a halide to iron, and the C–X bond is formed through a radical rebound following the activation of the iron cofactor by molecular oxygen.^10^ Similarly, the proposed mechanism of the recently discovered dicopper halogenase ApnU also invokes hydrogen atom abstraction by an O_2_-activated metallocofactor followed by a radical transfer of a directly coordinated halogen.^49^ We propose the involvement of an analogous diiron–chloro metallocofactor in the diiron halogenases and a mechanistic hypothesis involving C–H abstraction by a high-spin diiron–oxo species (Figure S21). Many aspects of this mechanistic hypothesis, specifically the formation of a di-Fe­(IV) intermediate, are similar to intermediate Q in soluble methane monooxygenase (sMMO).^20,50^ However, we propose that chlorination occurs via a chlorine transfer step resembling that of the mononuclear iron halogenases (Figure S21). This proposal could account for the reactivity of diiron halogenases. Moreover, this mechanism suggests that like the mononuclear nonheme radical halogenases, diiron halogenases may have evolved from hydroxylating diiron enzymes. This parallel evolution in biochemical logic may inform efforts to engineer halogenase activity in diiron hydroxylase enzymes, which has been explored in their mononuclear iron counterparts.^32,34,51^ Efforts to structurally characterize NocO and study the mechanism of diiron-catalyzed chlorination are ongoing. Additional insights into this halogenase family will continue to broaden our knowledge of enzymatic C–H functionalization in natural product biosynthesis.

## Supplementary Material



## Notes

Experimental details, primers used for plasmid construction, and supplemental LC/MS data (PDF)

## Notes

The following are the UniProt identifiers for the enzymes characterized in this study: NocL (A0A7S6RCM7), NocM (A0A7U3NLF1), and NocO (A0A7U3NLB0).

## Notes

This contribution is dedicated to the memory of Professor Christopher T. Walsh, an important mentor to E.P.B. and J.M.B., whose foundational work on halogenases in natural product biosynthesis inspired this study.

## Abbreviations

EIC: extracted ion chromatogram
LC/MS: liquid chromatography/mass spectrometry
MS: mass spectrometry
NADPH: nicotinamide adenine dinucleotide phosphate.

## References

[1] Pham J. V., Yilma M. A., Feliz A., Majid M. T., Maffetone N., Walker J. R., Kim E., Cho H. J., Reynolds J. M., Song M. C., Park S. R., Yoon Y. J. (2019). A Review
of the
Microbial Production of Bioactive Natural Products and Biologics. Frontiers in Microbiology.

[2] Fraley A. E., Sherman D. H. (2018). Halogenase Engineering and Its Utility
in Medicinal
Chemistry. Bioorg. Med. Chem. Lett..

[3] Wishart D. S., Feunang Y. D., Guo A. C., Lo E. J., Marcu A., Grant J. R., Sajed T., Johnson D., Li C., Sayeeda Z., Assempour N., Iynkkaran I., Liu Y., Maciejewski A., Gale N., Wilson A., Chin L., Cummings R., Le D., Pon A., Knox C., Wilson M. (2018). DrugBank 5.0: A Major Update to the DrugBank Database
for 2018. Nucleic Acids Res..

[4] Arndtsen B. A., Bergman R. G., Mobley T. A., Peterson T. H. (1995). Selective Intermolecular
Carbon–Hydrogen Bond Activation by Synthetic Metal Complexes
in Homogeneous Solution. Acc. Chem. Res..

[5] Crowe C., Molyneux S., Sharma S. V., Zhang Y., Gkotsi D. S., Connaris H., Goss R. J. M. (2021). Halogenases:
A Palette of Emerging
Opportunities for Synthetic Biology–Synthetic Chemistry and
C–H Functionalisation. Chem. Soc. Rev..

[6] Vaillancourt F. H., Yin J., Walsh C. T. (2005). SyrB2 in Syringomycin E Biosynthesis Is a Nonheme Fe­(II)
α-Ketoglutarate- and O2-Dependent Halogenase. Proc. Natl. Acad. Sci. U.S.A..

[7] Galonić D. P., Vaillancourt F. H., Walsh C. T. (2006). Halogenation of Unactivated Carbon
Centers in Natural Product Biosynthesis: Trichlorination of Leucine
during Barbamide Biosynthesis. J. Am. Chem.
Soc..

[8] Chang Z., Flatt P., Gerwick W. H., Nguyen V.-A., Willis C. L., Sherman D. H. (2002). The Barbamide Biosynthetic Gene Cluster: A Novel Marine
Cyanobacterial System of Mixed Polyketide Synthase (PKS)-Non-Ribosomal
Peptide Synthetase (NRPS) Origin Involving an Unusual Trichloroleucyl
Starter Unit. Gene.

[9] Vaillancourt F. H., Yeh E., Vosburg D. A., O’Connor S. E., Walsh C. T. (2005). Cryptic Chlorination
by a Non-Haem Iron Enzyme during Cyclopropyl Amino Acid Biosynthesis. Nature.

[10] Blasiak L. C., Vaillancourt F. H., Walsh C. T., Drennan C. L. (2006). Crystal Structure
of the Non-Haem Iron Halogenase SyrB2 in Syringomycin Biosynthesis. Nature.

[11] Galonić D. P., Barr E. W., Walsh C. T., Bollinger J. M., Krebs C. (2007). Two Interconverting
Fe­(IV) Intermediates
in Aliphatic Chlorination by the Halogenase CytC3. Nat. Chem. Biol..

[12] Matthews M. L., Krest C. M., Barr E. W., Vaillancourt F. H., Walsh C. T., Green M. T., Krebs C., Bollinger J. M. (2009). Substrate-Triggered Formation and
Remarkable Stability
of the C–H-Cleaving Chloroferryl Intermediate in the Aliphatic
Halogenase, SyrB2. Biochemistry.

[13] Matthews M.
L., Chang W., Layne A. P., Miles L. A., Krebs C., Bollinger J. M. (2014). Direct Nitration and Azidation of
Aliphatic Carbons by an Iron-Dependent Halogenase. Nat. Chem. Biol..

[14] Neugebauer M. E., Sumida K. H., Pelton J. G., McMurry J. L., Marchand J. A., Chang M. C. Y. (2019). A Family of Radical
Halogenases for the Engineering
of Amino-Acid-Based Products. Nat. Chem. Biol..

[15] Nakamura H., Schultz E. E., Balskus E. P. (2017). A New Strategy for Aromatic Ring
Alkylation in Cylindrocyclophane Biosynthesis. Nat. Chem. Biol..

[16] Nakamura H., Hamer H. A., Sirasani G., Balskus E. P. (2012). Cylindrocyclophane
Biosynthesis Involves Functionalization of an Unactivated Carbon Center. J. Am. Chem. Soc..

[17] Chanco E., Choi Y. S., Sun N., Vu M., Zhao H. (2014). Characterization
of the *N*-Oxygenase AurF from *Streptomyces
thioletus*. Bioorg. Med. Chem..

[18] Jasniewski A. J., Que L. (2018). Dioxygen Activation by Nonheme Diiron
Enzymes: Diverse Dioxygen Adducts, High-Valent Intermediates, and
Related Model Complexes. Chem. Rev..

[19] Rajakovich, L. J. ; Zhang, B. ; McBride, M. J. ; Boal, A. K. ; Krebs, C. ; Bollinger, J. M., Jr. 5.10 - Emerging Structural and Functional Diversity in Proteins With Dioxygen-Reactive Dinuclear Transition Metal Cofactors. In Comprehensive Natural Products III; Liu, H.-W. B. , Begley, T. P. , Eds.; Elsevier: 2020; pp 215–250. 10.1016/B978-0-12-409547-2.14864-4

[20] Banerjee R., Proshlyakov Y., Lipscomb J. D., Proshlyakov D. A. (2015). Structure
of the Key Species in the Enzymatic Oxidation of Methane to Methanol. Nature.

[21] Fox B. G., Lyle K. S., Rogge C. E. (2004). Reactions
of the Diiron Enzyme Stearoyl-Acyl
Carrier Protein Desaturase. Acc. Chem. Res..

[22] Small F. J., Ensign S. A. (1997). Alkene Monooxygenase
from *Xanthobacter* Strain Py2. J. Biol. Chem..

[23] Eusebio N., Rego A., Glasser N. R., Castelo-Branco R., Balskus E. P., Leão P. N. (2021). Distribution
and Diversity of Dimetal-Carboxylate
Halogenases in Cyanobacteria. BMC Genomics.

[24] Reis J. P. A., Figueiredo S. A. C., Sousa M. L., Leão P. N. (2020). BrtB Is
an *O*-Alkylating Enzyme That Generates Fatty Acid-Bartoloside
Esters. Nat. Commun..

[25] Lopez J. A. V., Petitbois J. G., Vairappan C. S., Umezawa T., Matsuda F., Okino T. (2017). Columbamides
D and E: Chlorinated Fatty Acid Amides from the Marine
Cyanobacterium *Moorea bouillonii* Collected in Malaysia. Org. Lett..

[26] Gutiérrez-del-Río I., Brugerolle de Fraissinette N., Castelo-Branco R., Oliveira F., Morais J., Redondo-Blanco S., Villar C. J., Iglesias M. J., Soengas R., Cepas V., Cubillos Y. L., Sampietro G., Rodolfi L., Lombó F., González S. M. S., López Ortiz F., Vasconcelos V., Reis M. A. (2020). Chlorosphaerolactylates A–D:
Natural Lactylates of Chlorinated Fatty Acids Isolated from the Cyanobacterium *Sphaerospermopsis* sp. LEGE 00249. J. Nat. Prod..

[27] Martins T. P., Glasser N. R., Kountz D. J., Oliveira P., Balskus E. P., Leão P. N. (2022). Biosynthesis
of the Unusual Carbon Skeleton of Nocuolin
A. ACS Chem. Biol..

[28] Glasser N. R., Cui D., Risser D. D., Okafor C. D., Balskus E. P. (2024). Accelerating the
Discovery of Alkyl Halide-Derived Natural Products Using Halide Depletion. Nat. Chem..

[29] Choi Y. S., Zhang H., Brunzelle J. S., Nair S. K., Zhao H. (2008). In Vitro Reconstitution
and Crystal Structure of P-Aminobenzoate *N*-Oxygenase
(AurF) Involved in Aureothin Biosynthesis. Proc.
Natl. Acad. Sci. U. S. A..

[30] Tosha T., Hasan M. R., Theil E. C. (2008). The Ferritin
Fe_2_ Site
at the Diiron Catalytic Center Controls the Reaction with O_2_ in the Rapid Mineralization Pathway. Proc.
Natl. Acad. Sci. U.S.A..

[31] Kurtz D. M. (1997). Structural
Similarity and Functional Diversity in Diiron–Oxo Proteins. JBIC.

[32] Neugebauer M. E., Kissman E. N., Marchand J. A., Pelton J. G., Sambold N. A., Millar D. C., Chang M. C. Y. (2022). Reaction Pathway Engineering Converts
a Radical Hydroxylase into a Halogenase. Nat.
Chem. Biol..

[33] Mitchell A. J., Zhu Q., Maggiolo A. O., Ananth N. R., Hillwig M. L., Liu X., Boal A. K. (2016). Structural Basis
for Halogenation by Iron- and 2-Oxo-Glutarate-Dependent
Enzyme WelO5. Nat. Chem. Biol..

[34] Mitchell A. J., Dunham N. P., Bergman J. A., Wang B., Zhu Q., Chang W., Liu X., Boal A. K. (2017). Structure-Guided
Reprogramming of a Hydroxylase To Halogenate Its Small Molecule Substrate. Biochemistry.

[35] Matthews M. L., Neumann C. S., Miles L. A., Grove T. L., Booker S. J., Krebs C., Walsh C. T., Bollinger J. M. (2009). Substrate Positioning Controls the Partition between
Halogenation and Hydroxylation in the Aliphatic Halogenase, SyrB2. Proc. Natl. Acad. Sci. U. S. A..

[36] Simurdiak M., Lee J., Zhao H. (2006). A New Class
of Arylamine Oxygenases: Evidence That
p-Aminobenzoate *N*-Oxygenase (AurF) Is a Di-Iron Enzyme
and Further Mechanistic Studies. ChemBioChem..

[37] Brown C. A., Remar G. J., Musselman R. L., Solomon E. I. (1995). Spectroscopic and
Electronic Structure Studies of Met-Hemerythrin Model Complexes: A
Description of the Ferric–Oxo Dimer Bond. Inorg. Chem..

[38] Münck, E. In Physical Methods in Bioinorganic Chemistry; University Science Books: 2000; p 287.

[39] Gütlich, P. ; Bill, E. ; Trautwein, A. X. Mössbauer Spectroscopy and Transition Metal Chemistry: Fundamentals and Applications; Springer Berlin Heidelberg: 2011.

[40] Vincent J. B., Olivier-Lilley G. L., Averill B. A. (1990). Proteins Containing Oxo-Bridged Dinuclear
Iron Centers: A Bioinorganic Perspective. Chem.
Rev..

[41] Atkin C. L., Thelander L., Reichard P., Lang G. (1973). Iron and Free Radical
in Ribonucleotide Reductase: Exchange of iron and Mössbauer
spectroscopy of the protein B2 Subunit of the *Escherichia
coli* enzyme. J. Biol. Chem..

[42] Lynch J. B., Juarez-Garcia C., Münck E., Que L. (1989). Mössbauer and EPR Studies of the Binuclear Iron Center in
Ribonucleotide Reductase from *Escherichia coli*. J. Biol. Chem..

[43] Wörsdörfer B., Conner D. A., Yokoyama K., Livada J., Seyedsayamdost M., Jiang W., Silakov A., Stubbe J., Bollinger J. M., Krebs C. (2013). Function of the Diiron Cluster of *Escherichia coli*Class Ia Ribonucleotide Reductase in Proton-Coupled
Electron Transfer. J. Am. Chem. Soc..

[44] Korboukh V.
K., Li N., Barr E. W., Bollinger J. M., Krebs C. (2009). A Long-Lived,
Substrate-Hydroxylating Peroxodiiron­(III/III) Intermediate in the
Amine Oxygenase, AurF, from *Streptomyces thioluteus*. J. Am. Chem. Soc..

[45] Makris T. M., Vu V. V., Meier K. K., Komor A. J., Rivard B. S., Münck E., Que L., Lipscomb J. D. (2015). An Unusual
Peroxo Intermediate of the Arylamine Oxygenase of the Chloramphenicol
Biosynthetic Pathway. J. Am. Chem. Soc..

[46] Knoot C. J., Kovaleva E. G., Lipscomb J. D. (2016). Crystal
Structure of CmlI, the Arylamine
Oxygenase from the Chloramphenicol Biosynthetic Pathway. J. Biol. Inorg. Chem..

[47] Kulik H. J., Blasiak L. C., Marzari N., Drennan C. L. (2009). First-Principles
Study of Non-Heme Fe­(II) Halogenase SyrB2 Reactivity. J. Am. Chem. Soc..

[48] Blanksby S. J., Ellison G. B. (2003). Bond Dissociation Energies of Organic Molecules. Acc. Chem. Res..

[49] Chiang C.-Y., Ohashi M., Le J., Chen P.-P., Zhou Q., Qu S., Bat-Erdene U., Hematian S., Rodriguez J. A., Houk K. N., Guo Y., Loo J. A., Tang Y. (2025). Copper-Dependent
Halogenase Catalyses Unactivated C–H Bond Functionalization. Nature.

[50] Cutsail G. E. I., Banerjee R., Zhou A., Que L., Lipscomb J. D., DeBeer S. (2018). High-Resolution
Extended X-Ray Absorption
Fine Structure Analysis Provides Evidence for a Longer Fe···Fe
Distance in the Q Intermediate of Methane Monooxygenase. J. Am. Chem. Soc..

[51] Papadopoulou A., Meierhofer J., Meyer F., Hayashi T., Schneider S., Sager E., Buller R. (2021). Re-Programming and Optimization of
a L-Proline Cis-4-Hydroxylase for the Cis-3-Halogenation of Its Native
Substrate. ChemCatChem..

