# Supplementary material for: Biochemical Studies of a Cyanobacterial Halogenase Support the Involvement of a Dimetal Cofactor
Source: Biochemistry. 2025 Apr 29;64(10):2173–80. doi: 10.1021/acs.biochem.4c00720 (PMC12101540; doi:10.1021/acs.biochem.4c00720)
Supplement: Supplementary file 1 [file bi4c00720_si_001.pdf]

Supporting Information

Biochemical studies of a cyanobacterial halogenase support the involvement of a dimetal cofactor

Michelle L. Wang,[1] Nathaniel R. Glasser,[1] Mrutyunjay A. Nair,[2] Carsten Krebs,[2,3] J. Martin Bollinger Jr.,[2]* Emily P. Balskus[1,4]**

[1]Department of Chemistry and Chemical Biology, Harvard University, Cambridge, Massachusetts 02138, United States

[2]Department of Chemistry, The Pennsylvania State University, University Park, Pennsylvania 16802, United States

[3]Department of Biochemistry and Molecular Biology, The Pennsylvania State University, University Park, Pennsylvania 16802, United States

[4]Howard Hughes Medical Institute, Harvard University, Cambridge, Massachusetts 02138, United States

*Correspondence to: balskus@chemistry.harvard.edu, ckrebs@psu.edu, jmb21@psu.edu

Table of Contents

Materials and general methods 4

Expression and purification of Noc enzymes from BL21(DE3) 4

General procedure for protein expression and purification: 4

Coexpression and purification of NocO variants with NocM from ArcticExpress(DE3) 6

***In vitro* biochemical assays** 7

Loading of dodecanoic acid on NocM by NocL 7

Chlorination of Sfp-loaded NocM–dodecanoyl thioester by NocO 7

Chlorination of NocL loaded NocM–12,12,12-d_3-dodecanoyl thioester by NocO 8

Chlorination of Sfp loaded NocM–dodecanoyl thioester by NocO or NocO mutants purified from ArcticExpress(DE3) 8

Trypsin digest and LC–MS for product formation 9

Ferrozine analysis for Fe^{2+} loading in ^{57}Fe-enriched NocO 9

Ferene-S analysis for iron quantification in ArcticExpress (DE3) NocO 9

Preparation of samples for Mössbauer spectroscopy 10

Mössbauer spectroscopy 10

Supplementary Tables and Figures 11

Table S1: Primers used for cloning *noc* genes (5' to 3') 11

Table S2: Primers used for site-directed mutagenesis of NocO 11

Table S3. List of CylC homologs screened for expression and solubility in *E. coli*. 12

Table S4. List of peptides targeted for MS/MS analysis. 12

Table S5. Calculated molar weights for purified proteins. 13

Table S7. Parameters used to simulate 4.2-K/53-mT Mössbauer spectrum of as-isolated NocO. 13

Table S8. A_{562} of triplicate solutions containing approximately 12.5 nmol ^{57}Fe-enriched NocO against a water blank from Ferrozine assay measuring Fe^{2+} content. 13

Figure S1. Full multiple sequence alignment of AurF (CAE02601.1), CylC (AFV96137.1), and CylC homologs BrtJ (AOH72611.1) and NocO (QOV09192.1). Putative active site residues are designated with an asterisk (*). 14

Figure S2. Sequence logo depicting residues that are greater than 90% conserved among 242 CylC homologs. Putative active site residues are denoted with asterisks (*) and the nonconserved glutamate is designated with a red asterisk. 15

Figure S3. AlphaFold2 predicted structure of the complex of CylB (blue) and CylC (green) superimposed onto the AurF crystal structure (PDB: 3CHT, teal) alongside the diiron–oxo cofactor from the AurF crystal structure superimposed onto the putative CylC active site. 16

Figure S4. The location of E283 (α-carbon highlighted in blue) differs between the original CylC homology model and AlphaFold structure predictions. In the AurF-based CylC homology model, E283 is located on a disordered loop to accommodate for the binding of an AurF-based diiron cofactor. In the AlphaFold3 predicted structure, E283 is found on an ordered helix that is part of the FDO-like α helix bundle and is located more distal from the predicted diiron binding site. This change may provide an open coordinate site for chloride binding. 16

Figure S5. Aligned AlphaFold2 predicted structures of the complex of CylB (blue) and CylC (green) and the complex of NocM (purple) and NocO (salmon). 17

Figure S6. Aligned AlphaFold3 predicted structures of BrtJ (magenta), ColD (orange), CylC (green), and NocO (salmon). 17

Figure S7. Aligned putative active site residues of diiron-chloro bound AlphaFold3 predicted structures of BrtJ (magenta), ColD (orange), CylC (green), and NocO (salmon). 18

Figure S8. Additional residues (labeled in red) identified within 5 Å of the right iron atom of the predicted diiron-chloro AlphaFold3 predicted structures of BrtJ (magenta), ColD (orange), CylC (green), and NocO (salmon). 18

Figure S9. SDS-PAGE depicting purified *N*-His-NocL (lane 2, 72460 Da), *N*-His-NocM (lane 3, 11918.04 Da), and copurified *N*-His-NocM/*N*-His-NocO (lane 4, 51214.23 Da). Samples were run on a 4–20%

Wedgewell Tris-Glycine mini precast gel alongside Precision Plus Dual Xtra Prestained Protein Standards (Bio-Rad) (lanes 1 and 5). 19
Figure S10. SDS-PAGE depicting ^{57}Fe enriched NocO, 5X diluted before addition into 2X Laemmli Buffer (Bio-Rad). Sample was run on a 10–20% Wedgewell Tris-Glycine mini precast gel alongside Precision Plus All Blue Prestained Protein Standards (Bio-Rad). 20
Figure S11. UV/Vis spectrum of a 20X diluted sample of as isolated ^{57}Fe-enriched NocO, with broad absorbance features at ~360 nm and ~410 nm. Inset: concentrated sample of ^{57}Fe-enriched NocO. 21
Figure S12. 4.2-K/53-mT Mössbauer spectrum of ^{57}Fe-enriched NocO reduced with excess sodium dithionite. The black arrows represent the high-energy lines of the diiron(III)-species that were reticent to reduction, as is quoted in Table S5. 21
Figure S13. Representative extracted ion chromatograms (EICs) depicting loading of dodecanoic acid onto NocM by NocL. Assays and negative controls were performed in triplicate. 22
Figure S14. Representative EICs depicting chlorination of NocM-dodecanoyl by purified NocO without a supplemented iron source *in vitro*. Assays and negative controls were performed in triplicate. 23
Figure S15. Representative EICs depicting lack of chlorination of NocM-dodecanoyl in the absence of NocO. Assays and negative controls were performed in triplicate. 24
Figure S16. Representative EICs depicting chlorination of NocM-dodecanoyl by as-isolated ^{57}Fe-enriched NocO *in vitro*. Assays and negative controls were performed in triplicate. 25
Figure S17. α-His Western blot depicting relative expression levels and solubility of WT NocO and the NocO variant NocO E129A when both are coexpressed with NocM in various BL21(DE3) derivatives. Lysates were prepared and clarified as described in above method and insoluble fractions were prepared via resuspension in 2% SDS. All samples were diluted 5X in water before addition into 2X Laemmli Buffer (Bio-Rad). Samples were run on a 10–20% Wedgewell Tris-Glycine mini precast gel (Invitrogen) alongside Precision Plus All Blue Protein Standards (Bio-Rad). SoluBL21(DE3) was sourced from Gelantis and Codon Plus RIL(DE3) was sourced from Agilent. 26
Figure S18. SDS-PAGE depicting NocM/NocO and respective variants as purified from ArcticExpress(DE3). Samples were run on a 10–20% Wedgewell Tris-Glycine mini precast gel alongside Precision Plus Dual Xtra Prestained Protein Standards (Bio-Rad). 27
Figure S19. SDS-PAGE depicting purified NocM/empty pET-Duet from ArcticExpress(DE3). Samples were run on a 10–20% Wedgewell Tris-Glycine mini precast gel alongside Precision Plus All Blue Protein Standards (Bio-Rad). 28
Figure S20. Iron content as determined by Ferene-S assay in a 10 mM sample of NocM and a buffer blank. Bars represent average of triplicate measurements (mean + SD). 29
Figure S21. Mechanistic hypothesis for chlorination by diiron halogenases. 29

Materials and general methods

Primers were supplied from Millipore Sigma or Genewiz from Azenta Life Sciences. Gene sequencing was performed by Genewiz from Azenta Life Sciences. Phusion Polymerase from New England Biolabs (NEB) was used for PCR amplification of target genes. Hifi DNA Assembly Master Mix from NEB was used for assembling protein expression vectors. KLD Enzyme Mix from NEB was used for site-directed mutagenesis. The QIAprep Spin Miniprep Kit and the QIAquick PCR purification kit were purchased from Qiagen. BL21(DE3) *E. coli* (lab stock), LOBSTR(DE3) *E. coli* (lab stock), and ArcticExpress(DE3) *E. coli* (Agilent) were used for protein expression. Protein concentrations were calculated using extinction coefficients predicted by the ExPASy ProtParam tool (https://web.expasy.org/protparam/) and measured absorbance at 280 nm or using a Bradford assay. Absorbances were measured using a Thermo Fisher Scientific NanoDrop 2000 UV–Vis spectrophotometer unless otherwise stated. Chemicals were purchased from Sigma-Aldrich or VWR, unless otherwise specified. All extracted ion chromatograms (EICs) obtained on the Agilent Q-TOF 6530 were performed with a 10 ppm error range, unless otherwise stated.

Expression and purification of Noc enzymes from BL21(DE3)

General procedure for protein expression and purification:

A corresponding plasmid was transformed into chemically competent BL21(DE3) *E. coli* cells. A freshly transformed colony was picked to inoculate 25 mL starter cultures in LB medium supplemented with respective antibiotic(s). The starter culture was incubated overnight at 37 °C with 190 r.p.m shaking. 20 mL of the overnight culture was used to inoculate 1 L of Terrific Broth medium (Research Products International, RPI) containing the appropriate antibiotic(s). The cultures were incubated at 37 °C with 200 r.p.m. shaking. At OD_{600} = 0.3–0.6, the cultures were cooled to 16 °C, and after 45 mins, protein expression was induced with 100 µM isopropyl β-D-1-thiogalactopyranoside (IPTG) (Teknova). The cultures were incubated for 18 h at 16 °C with 200 r.p.m. shaking.

Cells from 1 L of culture were pelleted by centrifugation (7,808 *xg* for 10 min) and resuspended in 20 mL of lysis buffer (20 mM HEPES, 300 mM NaCl, 30 mM imidazole, 10% glycerol, pH 8.0) and half an EDTA-free Pierce Protease Inhibitor Tablet (Thermo Fisher). The cells were lysed via sonication: 2 sec on, 8 sec off, for a total of 2 min on @ 25% amplitude. The lysate was supplemented with 50 µg/mL Deoxyribonuclease I (DNase I), mixed via pipetting, and allowed to stand on ice for an additional 5–10 mins. The lysate was clarified via centrifugation (15,000 *xg*) for 45 min and the cell debris was discarded. The supernatant was applied to a glass column containing ~2 mL of packed HisPur Co resin (Thermo Fisher) previously equilibrated in lysis buffer. The supernatant was allowed to slowly pass through the resin bed to allow proteins to bind. Unbound protein was discarded, and the resin was washed once with 20 mL of lysis buffer and once with 20 mL of wash 2 buffer (lysis buffer with 40 mM imidazole). Bound protein was eluted from the column in elution buffer (lysis buffer containing 250 mM imidazole) in five 5 mL fractions. SDS–PAGE analysis (4–20% Wedgewell Tris-Glycine mini precast gel, Invitrogen) was employed to determine the presence of protein in each fraction. Fractions containing the desired protein were combined and dialyzed against 1 L of dialysis buffer (20 mM

HEPES, 50 mM NaCl, 10% glycerol, pH 8.0) overnight at 4 °C. The dialyzed protein solution was concentrated using an Amicon 15 mL centrifugal concentrator with a membrane of corresponding MWCO cutoff. Protein concentration was determined via A_{280}, and the concentrated protein solutions were frozen with liquid nitrogen for storage at −80 °C.

<u>Specifications and deviations from the general method for each protein:</u>

<u>*N*-His$_6$-NocL</u>

pET15b was used for expression of *N*-His$_6$-*nocL*, and ampicillin (100 μg/mL) was used for antibiotic selection. The MWCO for centrifugal filters was 30 K. The protein was desalted on a PD-10 desalting column (Cytiva Life Sciences) according to manufacturer instructions. The procedure yielded approximately 1 mL of 2.5 mg/mL (32.3 μM) of *N*-His$_6$-NocL.

<u>*N*-His$_6$-NocM</u>

pCDF-Duet was used for expression of *N*-His$_6$-*nocM* and spectinomycin (50 μg/mL) was used for antibiotic selection. Expression cultures were grown at a scale of 2 L, and above volumes were scaled accordingly. Protein was purified on Ni-NTA resin (Qiagen). The MWCO for centrifugal filters was 3 K. The protein was desalted on a PD-10 desalting column. This procedure yielded approximately 1 mL of 7.4 mg/mL (620.1 μM) of *N*-His$_6$-NocM.

<u>Coexpressed *N*-His$_6$-NocM and *N*-His$_6$-NocO (natural abundance Fe)</u>

pET-Duet was used for expression of *N*-His$_6$-*nocO* and the plasmid was cotransformed with pCDF-Duet-NHis-*noc*M-emptyMCS2. Ampicillin and spectinomycin were used for antibiotic selection. 200 mg/L ferric ammonium citrate was supplemented into overnight starter culture and 100 mg/L ferric ammonium citrate was added at inoculation and at induction. Lysis buffer was also supplemented with 0.5 mg/mL lysozyme (RPI) and 0.5% (w/v%) β-D-*n*-octylthioglucopyranoside (Chem Impex International). Lysis was initiated via the resuspension of the cell pellet in the lysis mixture and the mixture was allowed to sit on ice for 15–20 mins to allow lysis to occur. 200 mg/L ferric ammonium citrate was added to the lysate prior to DNaseI treatment. The MWCO for centrifugal filters and dialysis cassette (Thermo Fisher) was 3 K and 3.5 K, respectively. This procedure yielded approximately 500 μL of 3.5 mg/mL of total protein, approximately 1.4 mg/mL (28 μM) was composed of *N*-His$_6$-NocO.

<u>Coexpressed *N*-His$_6$-NocM and *N*-His$_6$-NocO (^{57}Fe enriched)</u>

The plasmids were transformed into chemocompetent LOBSTR(DE3) cells, and a single colony was used to inoculate 100 mLs of M9 minimal media (12.8 g/L $Na_2HPO_4 \cdot 7\ H_2O$, 3.0 g/L KH_2PO_4, 1.0 g/L NH_4Cl, 0.5 g/L NaCl, 0.4% glucose, 10 μM $CaCl_2$, 200 mM $MgSO_4$) overnight culture with half the amount of antibiotics as described above. Expression cultures were grown on an 8L scale, and 25 mLs of the previously described cultures were used to inoculate 1L of M9 minimal media supplemented with 2 g/L of low iron and low salt casamino acids and half ampicillin and spectinomycin. 63.6 mg elemental ^{57}Fe (Isoflex) was dissolved 1 mL of 1M H_2SO_4 and brought to a final concentration of 500 mM of ^{57}Fe in M9 media. 200 μL of the ^{57}Fe solution was added to each liter of culture upon induction. Lysis buffer was supplemented with 100 μM ^{57}Fe and 0.25% *n*-dodecyl-β-D-maltoside (DDM, Anatrace) as the detergent source and 0.02% (w/v%) DDM was added to wash 2 buffer and elution buffer. Protein was purified on Ni-NTA resin. Protein was dialyzed against dialysis buffer containing 100 mM NaCl. The MWCO

for centrifugal filters and dialysis cassettes was 10 K. Total protein concentration was measured in triplicate by Bradford assay and relative concentration of NocO was determined using the software ImageJ using the "Gel Lane" function to estimate relative band intensities. This procedure yielded approximately 1.2 mL of 41.5 mg/mL total protein, approximately 24.0 mg/mL (467.5 μM) of which was composed of *N*-His_6-NocO.

Coexpression and purification of NocO variants with NocM from ArcticExpress(DE3)

pETDuet-solo-NHis_6-*nocO* or a corresponding mutant and pCDF-NHis_6-*nocM*-emptyMCS2 were cotransformed into chemically competent ArcticExpress(DE3) *E. coli* cells according to manufacturer instructions. The freshly transformed colonies were picked to inoculate 5 mL starter cultures in LB medium supplemented with 100 μg/mL ampicillin, 50 μg/mL spectinomycin, 20 μg/mL gentamycin, and 200 mg/L ferric ammonium citrate. The starter culture was incubated for 5 hours at 37 °C with 190 r.p.m shaking. After 5 hours, the starter culture was spun down at 4000 r.p.m. and the supernatant was decanted. The cell pellet was resuspended in 5 mL of M9 medium, and 1 mL of this resuspension was used to inoculate 50 mL of M9 medium with ampicillin, spectinomycin, gentamycin, and ferric ammonium citrate. This culture was allowed to grow overnight at 37 °C with 190 r.p.m shaking. 25 mL of the M9 overnight culture was used to inoculate 1 L of M9 medium containing ampicillin, spectinomycin, gentamycin, and 100 mg/L ferric ammonium citrate. The cultures were incubated at 37 °C with 200 r.p.m. shaking. At OD_{600} = 0.3–0.6, the cultures were cooled to 16 °C, and after 45 mins, the protein expression was induced with 100 μM IPTG and the cultures were supplemented with an additional 100 mg/L ferric ammonium citrate. The cultures were incubated for 18 h at 16 °C with 200 r.p.m. shaking.

Cells from 1 L of culture were pelleted by centrifugation (6000 r.p.m. for 10 min) and resuspended in 20 mL of lysis buffer (20 mM HEPES, 300 mM NaCl, 10 mM imidazole, 10% glycerol, pH 8.0) supplemented with 0.5 mg/mL lysozyme, 0.5% (w/v%) β-D-*n*-octylthioglucopyranoside, and half an EDTA-free Pierce Protease Inhibitor Tablet. The cells were resuspended in the lysis buffer mixture and were allowed to sit on ice for 15–20 mins to allow lysis to occur. The lysate was supplemented with 200 mg/L ferric ammonium citrate and 50 μg/mL DNase I, mixed via pipetting, and allowed to stand on ice for an additional 5–10 mins. The lysate was clarified via centrifugation (15,000 *xg*) for 45 mins and the cell debris was discarded. The supernatant was applied to a glass column containing ~2 mL of packed Ni-NTA resin previously equilibrated in lysis buffer. The supernatant was allowed to slowly pass through the resin bed to allow proteins to bind. Unbound protein was discarded, and the NTA resin was washed once with 40 mL of ATP wash buffer (lysis buffer containing 30 mM imidazole, 30 mM $MgCl_2$, and 15 mM ATP). Bound protein was eluted from the column in elution buffer (lysis buffer containing 250 mM imidazole) in five 5 mL fractions. SDS–PAGE analysis (4–20% Wedgewell Tris-Glycine mini precast gel) was employed to determine the presence of protein in each fraction. Fractions containing the desired protein were combined and dialyzed against 1 L of dialysis buffer (20 mM HEPES, 150 mM NaCl, 10% glycerol, pH 8.0) overnight at 4 °C. The dialyzed protein solution was concentrated using an Amicon 15 mL centrifugal concentrator with a 3 K MWCO membrane. Protein concentration was determined via Bradford Assays (average of triplicate measurements), and the concentrated protein solutions were frozen with liquid nitrogen

for storage at −80 °C.[a] In order to determine the concentration of *N*-His$_6$-NocO or a corresponding variant in solution, aliquots of concentrated protein were diluted 5X and 10X before addition into 2X Laemmli buffer (Bio-Rad) and subjected to SDS-PAGE. The relative band intensities of the most well resolved lanes were measured using the software ImageJ, and the proportion of NocO's peak area was scaled to the total protein concentration and converted to molarity using the molar masses described in Table S5.

An *E. coli* coexpression strain lacking NocO (negative control) was constructed by cotransforming chemically competent ArcticExpress(DE3) *E. coli* cells with pCDF-NHis$_6$-*nocM*-emptyMCS2 and an empty pETDuet vector.

In vitro biochemical assays

All reactions were carried out in a final volume of 50 µL. Purified enzyme aliquots were stored in enzyme storage buffers as described above. Stock solutions for Spinach Ferredoxin and Spinach Ferredoxin reductase were prepared in reaction buffer (20 mM HEPES, 50 mM NaCl, 10% glycerol). All *in vitro* reactions, including negative controls, were performed in triplicate unless otherwise specified.

<u>Loading of dodecanoic acid on NocM by NocL</u>

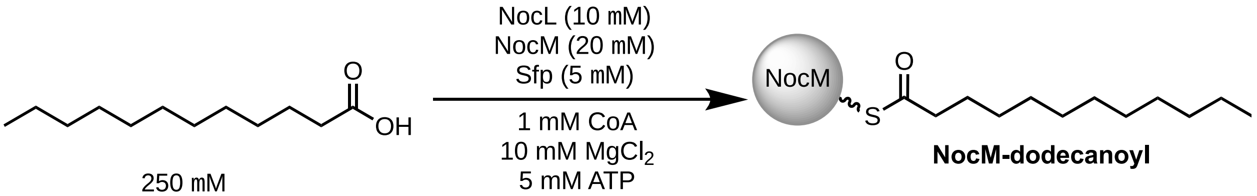

Scheme S1: Reaction scheme of loading of dodecanoic acid onto NocM by NocL

A 50 µL solution containing Sfp (5 µM), $MgCl_2$ (10 mM), ATP (5 mM, RPI), Coenzyme A (1 mM, RPI), and dodecanoic acid (250 µM in MeOH) was combined with NocL (10 µM) and NocM (20 µM) and incubated at room temperature for 3 h. Reaction mixtures were then frozen and stored at –80 °C until analysis.

Negative controls were reactions lacking NocL, which was replaced with an equivalent volume of exchange buffer as described above.

<u>Chlorination of Sfp-loaded NocM–dodecanoyl thioester by NocO</u>

In a 50 µL solution containing a final concentration of 20 mM HEPES and 50 mM NaCl, Sfp (5 µM), $MgCl_2$ (1 mM), Spinach Ferredoxin (100 µg/mL), Spinach Ferredoxin Reductase (100 mU/mL), TCEP (1 mM), dodecanoyl-CoA (500 µM), ammonium iron(II) sulfate hexahydrate (20 µM, BL21(DE3) only), NADPH (1 mM) were combined with 12.5 µL of copurified NocM/NocO from BL21(DE3) (approximately 7 µM NocO with 43 µM of NocM) or LOBSTR(DE3) (approximately 17 µM of ^{57}Fe-enriched NocO with 37 µM of NocM) incubated

---

[a] Samples used for H132A were combination of two different 1 L growths due to low yield.

at room temperature for 18 h. Reaction mixtures were then frozen and stored at –80 °C until analysis.

For full reactions with as-purified protein, the ferrous ammonium sulfate was replaced with an equivalent volume of water. For negative controls lacking NADPH, NADPH was replaced with an equivalent volume of water. For negative controls using purified NocM, 40 µM of NocM was used in place of the protein mixture.

Chlorination of NocL loaded NocM–12,12,12-*d*$_3$-dodecanoyl thioester by NocO

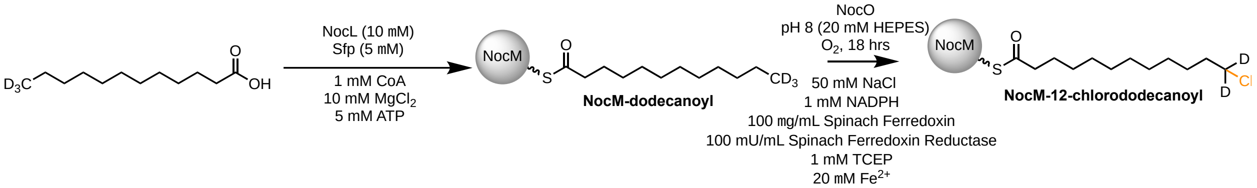

Scheme S2: Reaction scheme for chlorination of terminally deuterated NocM-dodecanoyl thioester

In a 50 µL solution containing a final concentration of 20 mM HEPES and 50 mM NaCl, Sfp (5 µM), $MgCl_2$ (10 mM), Spinach Ferredoxin (100 µg/mL), Spinach Ferredoxin Reductase (100 mU/mL), TCEP (1 mM), 12,12,12-*d*$_3$-dodecanoic acid (500 µM in MeOH), ferrous ammonium sulfate (20 µM), NADPH (1 mM), Coenzyme A (1 mM), and ATP (5 mM) were combined with 12.5 µL of copurified NocM/NocO (from BL21(DE3)) and NocL (10 µM) incubated at room temperature for 18 h. Reaction mixtures were then frozen and stored at –80 °C until analysis.

For negative controls lacking NADPH, NADPH was replaced with an equivalent volume of water. Full reactions and negative controls were performed in duplicate.

Chlorination of Sfp loaded NocM–dodecanoyl thioester by NocO or NocO mutants purified from ArcticExpress(DE3)

In a 50 µL solution containing a final concentration of 20 mM HEPES and approximately 50 mM NaCl, Sfp (5 µM), $MgCl_2$ (1 mM), Spinach Ferredoxin (100 µg/mL), Spinach Ferredoxin Reductase (100 mU/mL), TCEP (1 mM), dodecanoyl-CoA (500 µM), and NADPH (1 mM) were combined with the protein mixture containing coexpressed NocM/NocO from ArcticExpress(DE3) to a final concentration of 3 mg/mL of total protein[b] and incubated at room temperature for 18 h. Reaction mixtures were then frozen and stored at –80 °C until analysis.

For negative controls lacking redox components, Spinach Ferredoxin and Spinach Ferredoxin Reductase were replaced with equivalent volumes of reaction buffer and NADPH was replaced with an equivalent volume of water. For negative controls using copurified empty pETDuet/NocM, 40 µM of NocM combined with 20 µM of ammonium iron(II) sulfate hexahydrate was used in place of the protein mixture.

[b] Assays with H132A were performed at 2.5 mg/mL total protein.

Trypsin digest and LC–MS for product formation

For analysis of product formation, reaction mixtures were thawed on ice and 12.5 µL of the reaction mixture was combined with 12.5 µL of 200 mM ammonium biocarbonate containing 2 mM TCEP and 25 µL of 100 ng/µL sequencing grade trypsin (Promega). The trypsin digest was incubated at 37 °C for 2 hours. The resulting mixture was spun down at 16,100 *xg* for 5 mins to remove any precipitates and 25 µL of the mixture was collected for analysis by LC–MS.

An Agilent Q-TOF 6530 equipped with a Dual AJS ESI source was used for LC–MS analysis. A Phenomenex Aeris Widepore C18 column (3.6 µm, 150 x 4.6 mm) flowing at a rate of 0.5 mL/min was used. Solution A was H_2O + 0.1% formic acid, and Solution B was MeCN + 0.1% formic acid. The LC method was: 5% Solution B for 5 min; 5% to 95% Solution B over 21 min, 95% Solution B for 1 min, 95% to 5% over 1 min, and 5% for 7 min. The following parameters were used for the Q-TOF: Gas Temp 325 °C, Drying Gas 10 L/min, Nebulizer 45 psi, Sheath Gas Temp 275 °C, Sheath Gas Flow 11 L/min, VCap 4000 V, Nozzle Voltage 1000 V.

Ferrozine analysis for Fe^{2+} loading in ^{57}Fe-enriched NocO

A solution containing approximately 12.5 nmol of ^{57}Fe-enriched NocO was denatured with 0.2 volumes of 50% trifluoroacetic acid in water and centrifuged at maximum speed at room temperature to remove protein precipitates. The supernatants were removed and combined with enough water to bring to a volume of 500 µL. A blank was created using 500 µL of water. Each sample and the blank were combined with 20 µL of 75 mM ascorbic acid, 20 µL of 1 mM ferrozine (3-(2-pyridyl)-5,6-diphenyl-1,2,4-triazine-4′,4″-disulfonic acid sodium salt), and 120 µL of saturated ammonium acetate. The mixtures were briefly vortexed and absorbance at 562 nm was measured on an Agilent Cary UV/visible 3500 spectrometer against the water blank. Iron loading was calculated under the assumption that A_{562}=1 was equivalent to 25 nmol of Fe^{2+}.

Ferene-S analysis for iron quantification in ArcticExpress (DE3) NocO

To estimate the relative concentration of NocO in protein samples prepared from ArcticExpress, SDS-PAGE images were analyzed using the software ImageJ using the "Gel Lane" function. The area of major peaks of the gels were determined and the proportion of areas was scaled to the total protein concentration determined previously by a Bradford assay. Once the approximate concentration of NocO was determined, the protein mixture was diluted in storage buffer to contain approximately 10 µM NocO. For the negative control (empty pET-Duet/*N*-His-NocM), 10 µM NocM was used.

The iron content of a 100 µL solution containing approximately 10 µM of NocO was determined using Ferene (3-(2-pyridyl)-5,6-di(2-furyl)-1,2,4-triazine-5,5″-disulfonic acid disodium salt), according to a previously published procedure,[1] with the only difference being that the standard curve was prepared with ammonium iron(II) sulfate hexahydrate. After the addition of the Ferene reagent and thorough vortexing, 120 µL of each assay mixture was pipetted into the wells of a clear, flat bottom 96-well plate. The plate was spun down briefly at 4000 r.p.m. to remove bubbles and the absorbance at 592 nm was measured using a BioTek Synergy Neo2 plate reader.

Preparation of samples for Mössbauer spectroscopy

A Mössbauer sample cup was placed into a scintillation vial and 200 µL of ^{57}Fe-enriched NocO, prepared as described above, was carefully pipetted into the cup, avoiding bubbles. The scintillation vial was capped and lowered slowly into liquid nitrogen and the sample cup was given several minutes to completely freeze. Once frozen, the cup was removed from the scintillation vial and placed into another precooled vial. The vial was capped and placed into a storage dewar until sample analysis.

For the sodium dithionite reduced sample, 200 µL of aerobically purified ^{57}Fe-enriched NocO was frozen in liquid nitrogen and cycled into a glovebox. The protein was allowed to thaw at 4 °C, at which point it was placed into a 10K MWCO centrifugal spin filter and concentrated to a volume of 25-50 µL. Enough buffer (25 mM HEPES, 100 mM NaCl, 2 mM sodium dithionite, pH 7.5) was added to bring the total volume to 200 µL and the process was repeated 3 times. The final sample was spun down at 15,100 *xg* to remove precipitates and then pipetted into a Mössbauer sample cup in a scintillation vial. The vial was removed from the glovebox and frozen immediately and stored as described above.

Mössbauer spectroscopy

Mössbauer spectra were acquired on a spectrometer from SEECO (Edina, MN) equipped with a Janis SVT-400 variable temperature cryostat. The reported isomer shift is relative to the centroid of the spectrum of α-iron foil acquired at room temperature. The spectra were acquired at 4.2 K in the presence of 53 mT external magnetic field applied parallel to the direction of propagation of the γ-beam. Simulations of the Mössbauer spectra were carried out using the WMOSS spectral analysis software from SEECO (www.wmoss.org, SEE Co., Edina, MN).

Supplementary Tables and Figures

Table S1: Primers used for cloning *noc* genes (5' to 3')

Gene	Forward Primer	Reverse Primer
nocL	CATCATCACAGCAGCGGCATGAATGTTCGTTTGACCGATT	CTTTGTTAGCAGCCGGATCCTCTTATTGAAGTGCATTAGCTTTTTCAC
nocM	CAGCCATCACCATCATCACCACGGCAGCACGCAACTTTTTGAAGACAAC	CAAGCTTGTCGACCTGCAGTTATTAGTTTTCCTCAGCCACATATTC
nocO	CAGCCATCACCATCATCACCACGGCAGCATTACTAGCAGTAATACTTCACC	GTAGACGAGTCCATGTGCTTATTAAATTGCTATAGAAAACTGATTAAAGG

Table S2: Primers used for site-directed mutagenesis of NocO

Mutant	Forward Primer	Reverse Primer
E94A	CGACTGAGCTAATTCATTACAACAC	CACTGTCAGCAACTCTCTTG
Q128A	GCGGAACGTCAACATATTCATGCT	GTAAGTTTCATGAGCTAACAGTTCC
E129A	CGCGTCAACATATTCATGCTTTTTCTC	CTTGGTAAGTTTCATGAGCTAAC
H132A	ATTCATGCTTTTTCTCAAATAAATTATAAAACTACTAAAGC	CGCTTGACGTTCTTGGTAAGTTTCATG
E252A	CGTTTGGTATACATTGCTACTTCAAAAAAATGC	CATGGTTTTTCAATAGTAGATTAGCC
H279A	GCGTTTTTAGATGAAGCTTTTCATACAACC	ATAGTGAGATATAGCTGTTGGAC
D282A	CGGAAGCTTTTCATACAACCACT	CTAAAAAATGATAGTGAGATATAGCTG
E283A	CGGCTTTTCATACAACCACTTCTC	CATCTAAAAAATGATAGTGAGATATAGC
H286A	GCGACAACCACTTCTCTATTTCTTGG	AAAAGCTTCATCTAAAAAATGATAGTG

Table S3. List of CylC homologs screened for expression and solubility in *E. coli*.

CylC homolog	Accession Number	Organism
CylC	AFV96137.1	*Nostoc punctiforme* ATCC 29412
BrtJ	AKV71855	*Synechocystis salina* LEGE 06155
ColD	WP_198954205.1	*Moorena bouilloni* PNG
CylC2	unpublished	*Nostoc* sp. UIC 10022A
Npun_F3358	WP_012409761.1	*Nostoc punctiforme* ATCC 29133
NJK50442	NJK50442.1	Candidatus *Gracillibacteria* bacterium (metagenome)
LEGE_91341	WP_193956964.1	*Microcystis aeruginosa* sp. LEGE 91341
LEGE_12446_2	WP_193912923.1	*Desmonostoc muscorum* LEGE 12446
sll0266	AGF52187.1	*Synechocystis* sp. PCC 6803
cyl0266	unpublished	*Cylindrospermum licheniforme* ATCC 29412
F559_RS26160	WP_051319191.1	*Chitinimonas koreensis* DSM 17726
DFR29_RS120425	WP_133818525.1	*Tahibacter aquaticus* DSM 21667

Table S4. List of peptides targeted for MS/MS analysis.

Peptide species	Chemical formula	$[M+3H]^{3+}$ (*m/z*)
NocM–dodecanoyl thioester	$C_{120}H_{195}N_{26}O_{38}PS$	891.4601
NocM–12-chlorododecanoyl thioester	$C_{120}H_{194}ClN_{26}O_{38}PS$	902.7804
NocM–12,12,12-d_3-dodecanoyl thioester	$C_{120}H_{192}D_3N_{26}O_{38}PS$	892.4664
NocM–12-chloro-12,12-d_2-dodecanoyl thioester	$C_{120}H_{192}D_2ClN_{26}O_{38}PS$	903.4513

Table S5. Calculated molar weights for purified proteins.

Protein	Molar Mass (g/mol)
N-His-NocL	72460
N-His-NocM	11918.04
N-His-NocO (wild-type)	51214.23
N-His NocO H132A, H279A, H286A	51148.16
N-His NocO E94A, E129A, E252A, E283A	51156.19
N-His NocO D282A	51170.22
N-His NocO Q128A	51157.17

Table S6. Total protein yields and approximate concentration of NocO or the respective NocO variant post-purification from ArcticExpress(DE3).

Variant	Total protein (mg/mL)	[NocO] (μM) in solutions	[NocO] (μM) used in assays*
WT NocO	15.7	28.6	5.5
E94A	20.2	31.4	4.7
Q128A	16.1	22.2	4.1
E129A	16.0	20.1	3.8
H132A	11.1	19.1	4.3
E252A	12.0	19.9	5.0
H279A	15.4	21.9	4.3
D282A	12.9	21.9	5.1
E283A	16.7	26.4	4.7
H286A	14.5	23.2	4.8

*conditions described in procedure above

Table S7. Parameters used to simulate 4.2-K/53-mT Mössbauer spectrum of as-isolated NocO.

Species	δ (mm/s)	ΔE_Q (mm/s)	Γ (mm/s)	Area (%)
diferric 1 (blue)	0.53	0.9	0.33	50
diferric 2 (red)	0.49	1.44	0.31	23
diferric 3 (yellow)	0.53	2.15	0.25	17

Table S8. A_{562} of triplicate solutions containing approximately 12.5 nmol ^{57}Fe-enriched NocO against a water blank from Ferrozine assay measuring Fe^{2+} content.

Replicate	A_{562}	Fe^{2+} (nmol)
1	0.577	14.43
2	0.588	14.70
3	0.592	14.80

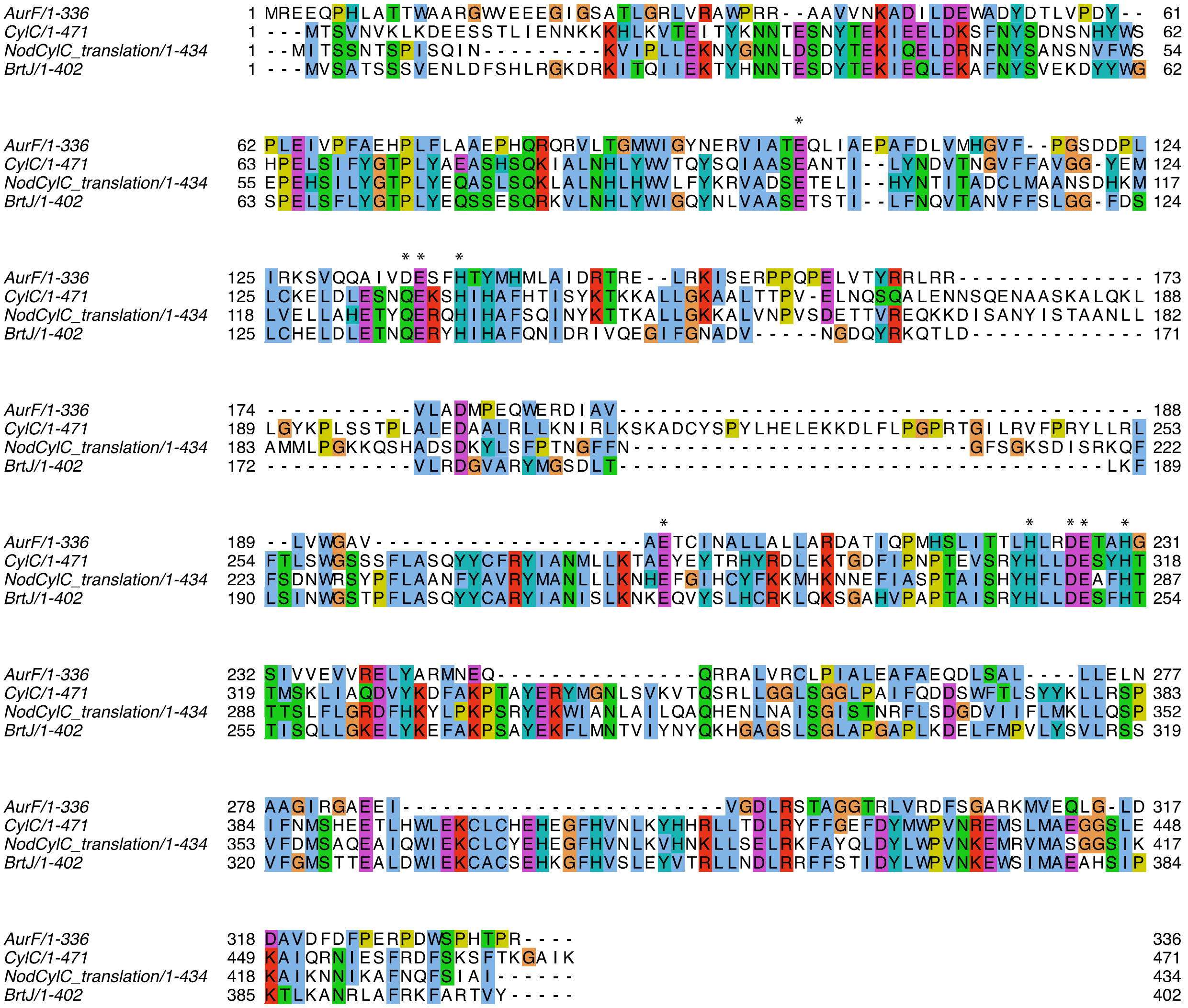

Figure S1. Full multiple sequence alignment of AurF (CAE02601.1), CylC (AFV96137.1), and CylC homologs BrtJ (AOH72611.1) and NocO (QOV09192.1). Putative active site residues are designated with an asterisk (*).

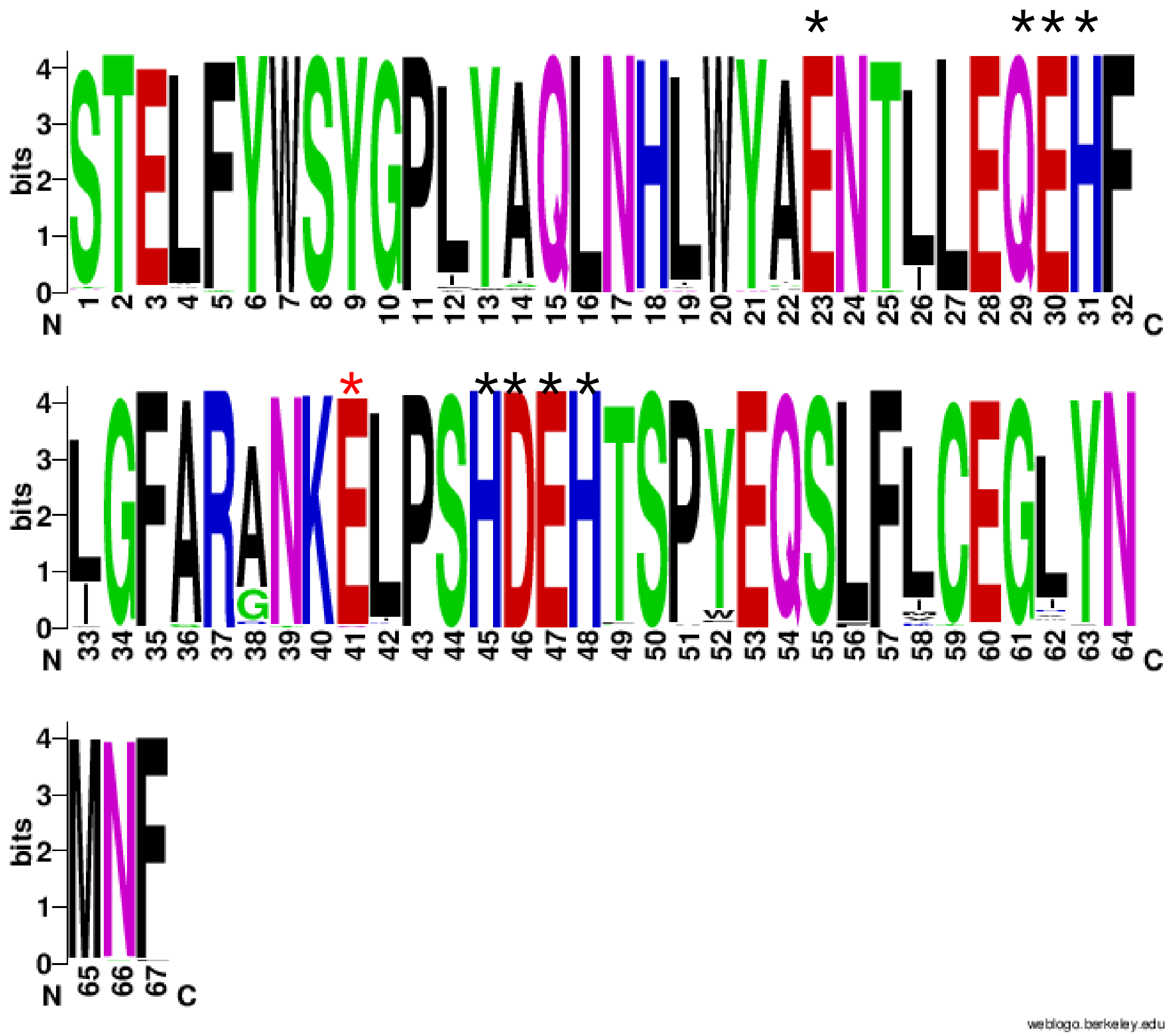

Figure S2. Sequence logo depicting residues that are greater than 90% conserved among 242 CylC homologs. Putative active site residues are denoted with asterisks (*) and the nonconserved glutamate is designated with a red asterisk.

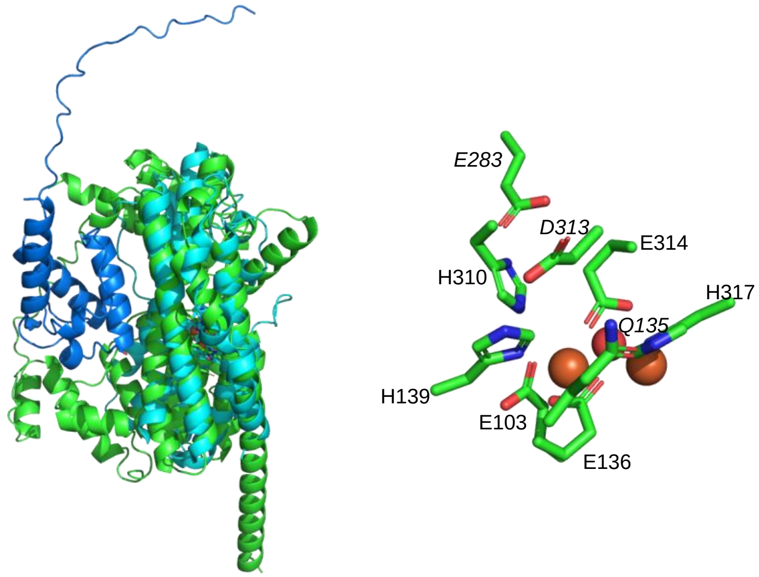

Figure S3. AlphaFold2 predicted structure of the complex of CylB (blue) and CylC (green) superimposed onto the AurF crystal structure (PDB: 3CHT, teal) alongside the diiron–oxo cofactor from the AurF crystal structure superimposed onto the putative CylC active site.

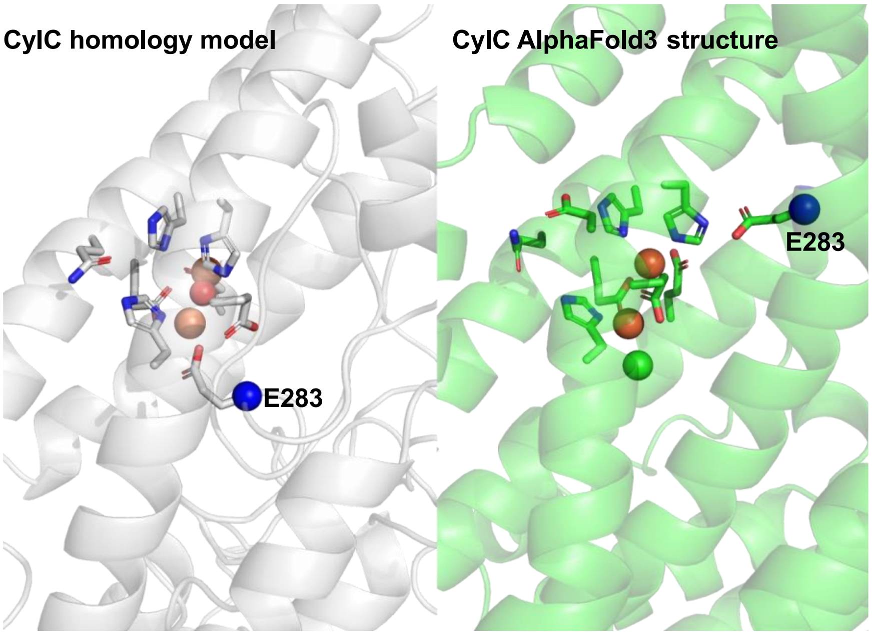

Figure S4. The location of E283 (α-carbon highlighted in blue) differs between the original CylC homology model and AlphaFold structure predictions. In the AurF-based CylC homology model, E283 is located on a disordered loop to accommodate for the binding of an AurF-based diiron cofactor. In the AlphaFold3 predicted structure, E283 is found on an ordered helix that is part of the FDO-like α-helix bundle and is located more distal from the predicted diiron binding site. This change may provide an open coordinate site for chloride binding.

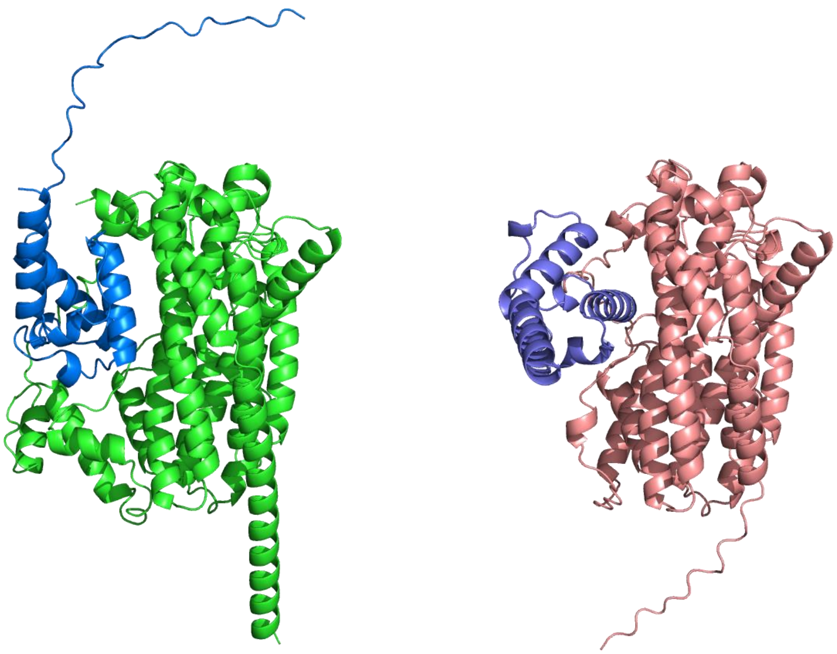

Figure S5. Aligned AlphaFold2 predicted structures of the complex of CylB (blue) and CylC (green) and the complex of NocM (purple) and NocO (salmon).

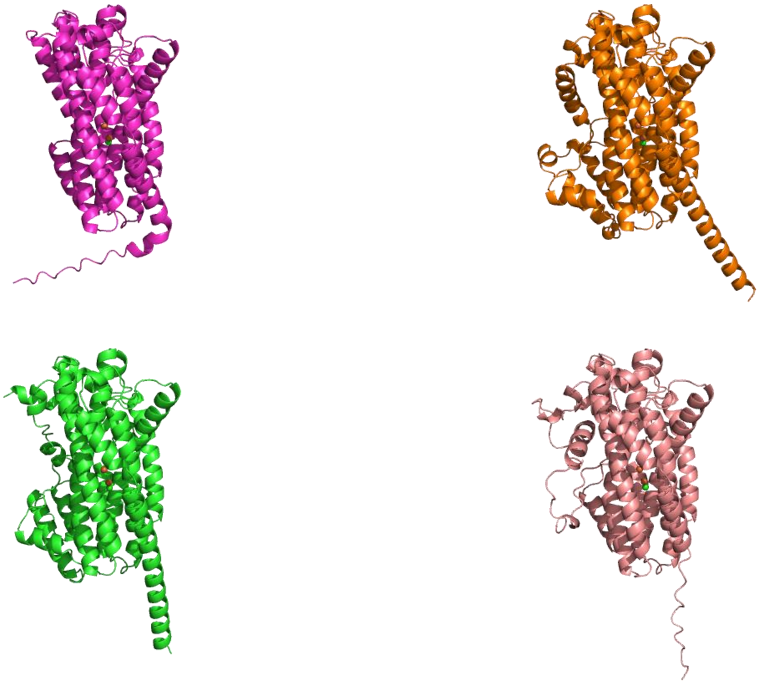

Figure S6. Aligned AlphaFold3 predicted structures of BrtJ (magenta), ColD (orange), CylC (green), and NocO (salmon).

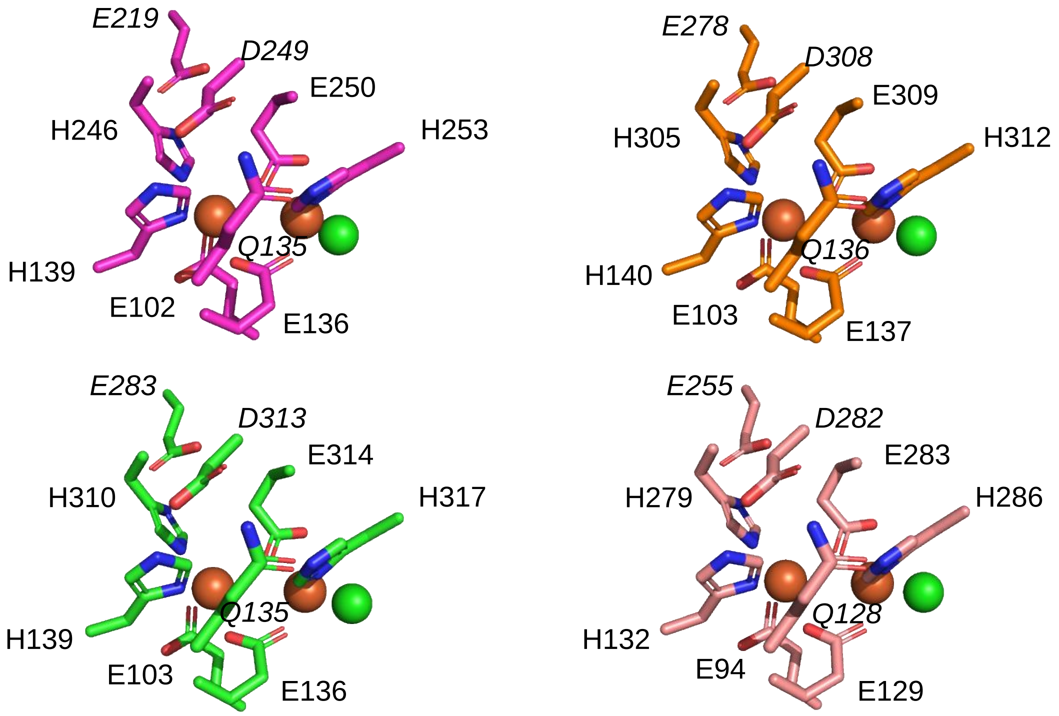

Figure S7. Aligned putative active site residues of diiron-chloro bound AlphaFold3 predicted structures of BrtJ (magenta), ColD (orange), CylC (green), and NocO (salmon).

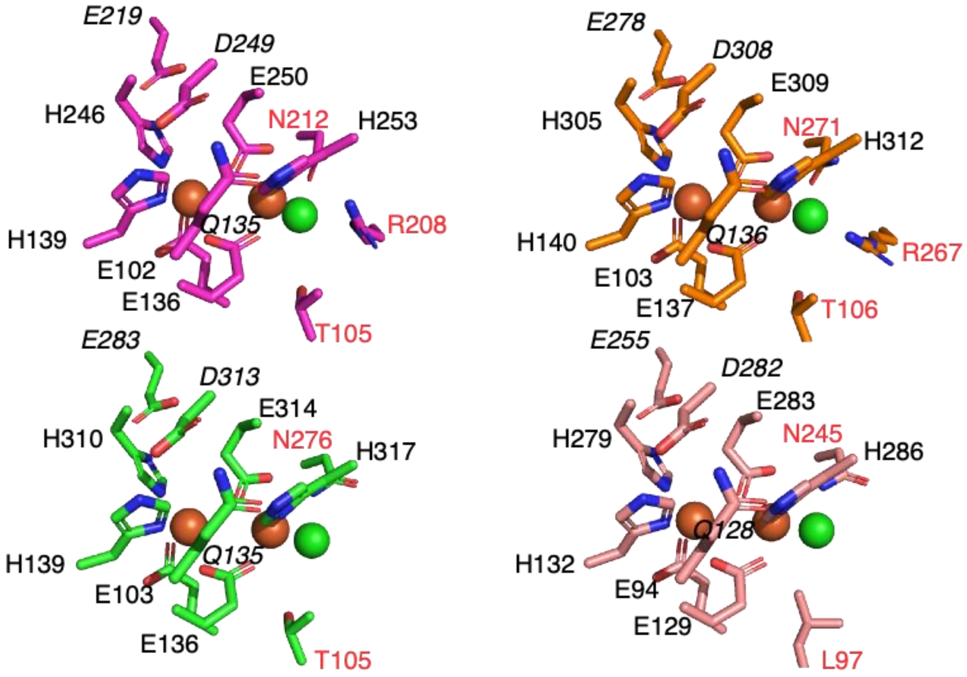

Figure S8. Additional residues (labeled in red) identified within 5 Å of the right iron atom of the predicted diiron-chloro AlphaFold3 predicted structures of BrtJ (magenta), ColD (orange), CylC (green), and NocO (salmon).

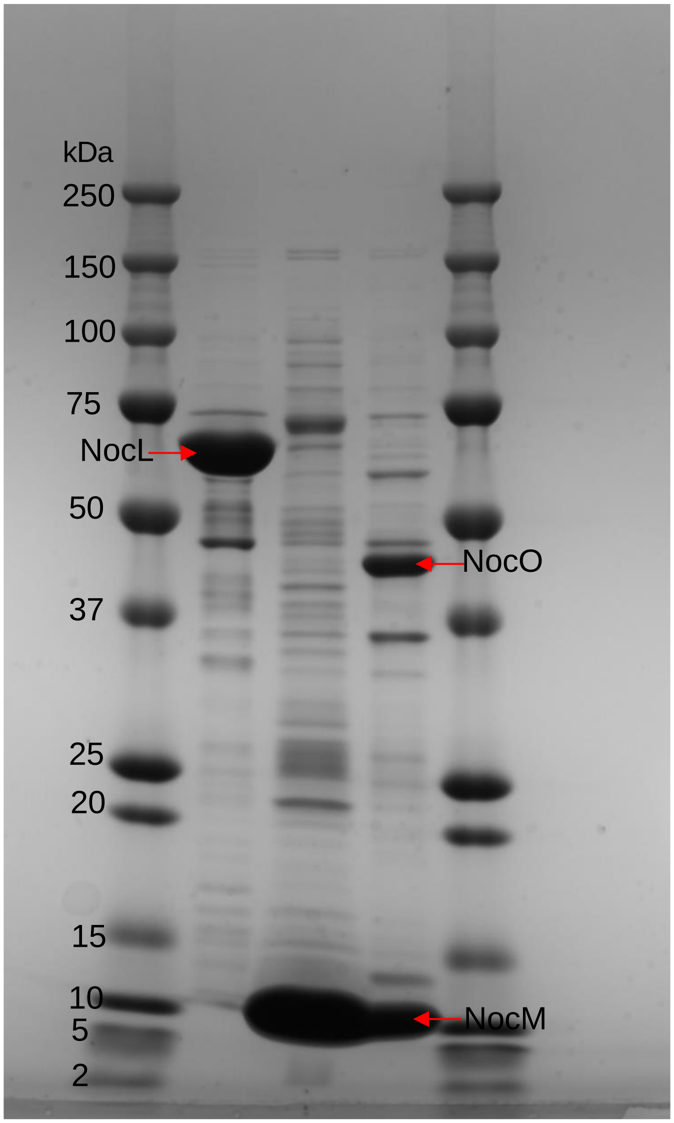

Figure S9. SDS-PAGE depicting purified *N*-His-NocL (lane 2, 72460 Da), *N*-His-NocM (lane 3, 11918.04 Da), and copurified *N*-His-NocM/*N*-His-NocO (lane 4, 51214.23 Da). Samples were run on a 4–20% Wedgewell Tris-Glycine mini precast gel alongside Precision Plus Dual Xtra Prestained Protein Standards (Bio-Rad) (lanes 1 and 5).

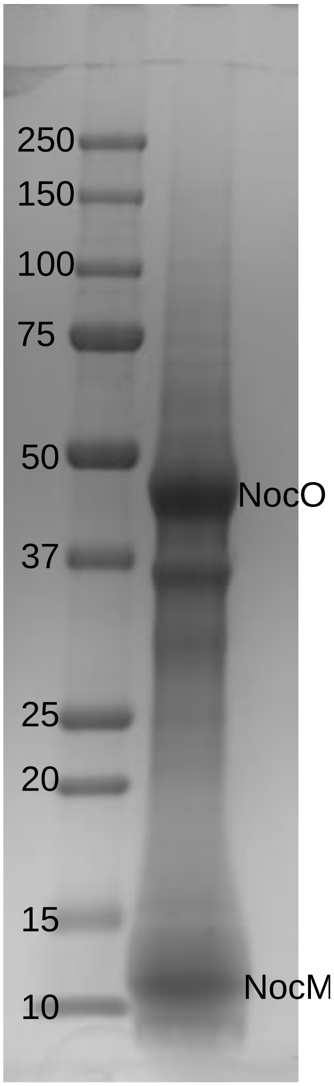

Figure S10. SDS-PAGE depicting ^{57}Fe enriched NocO, 5X diluted before addition into 2X Laemmli Buffer (Bio-Rad). Sample was run on a 10–20% Wedgewell Tris-Glycine mini precast gel alongside Precision Plus All Blue Prestained Protein Standards (Bio-Rad).

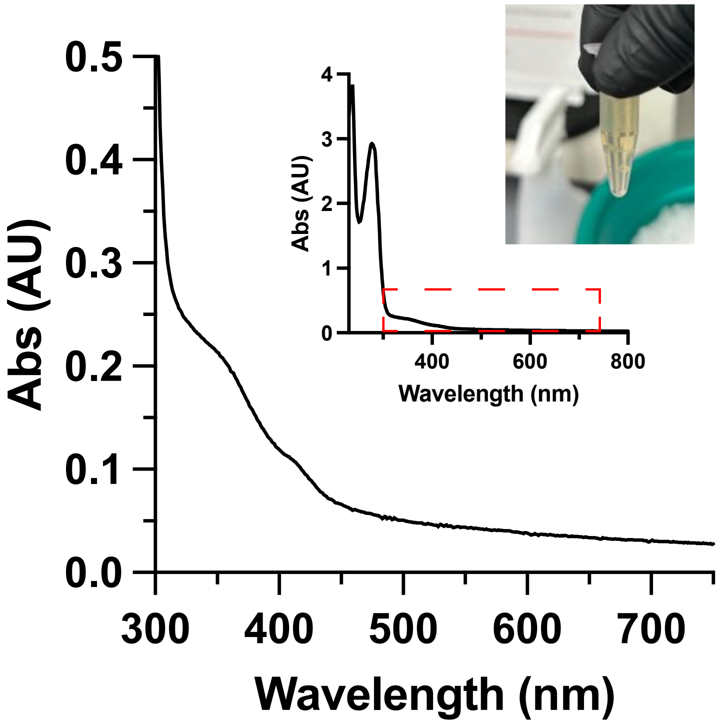

Figure S11. UV/Vis spectrum of a 20X diluted sample of as isolated ^{57}Fe-enriched NocO, with broad absorbance features at ~360 nm and ~410 nm. Inset: concentrated sample of ^{57}Fe-enriched NocO.

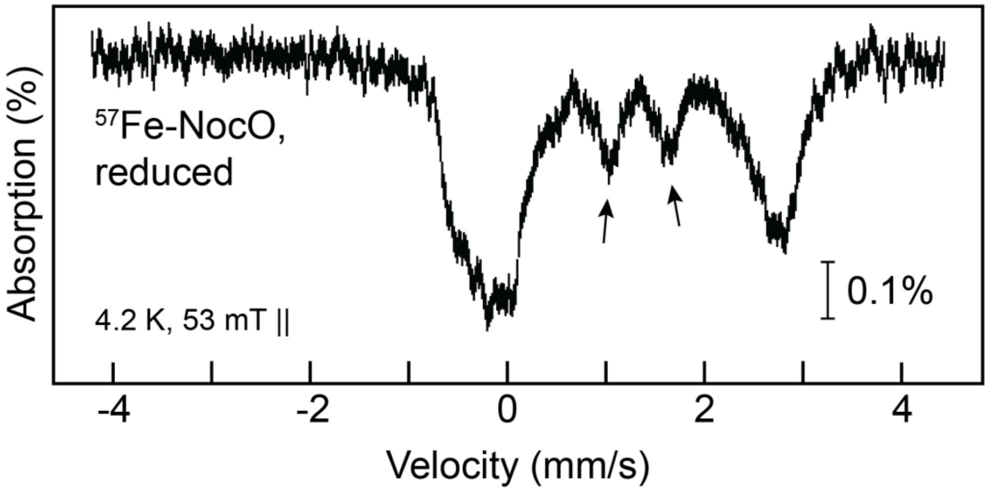

Figure S12. 4.2-K/53-mT Mössbauer spectrum of ^{57}Fe-enriched NocO reduced with excess sodium dithionite. The black arrows represent the high-energy lines of the diiron(III)-species that were reticent to reduction, as is quoted in Table S5.

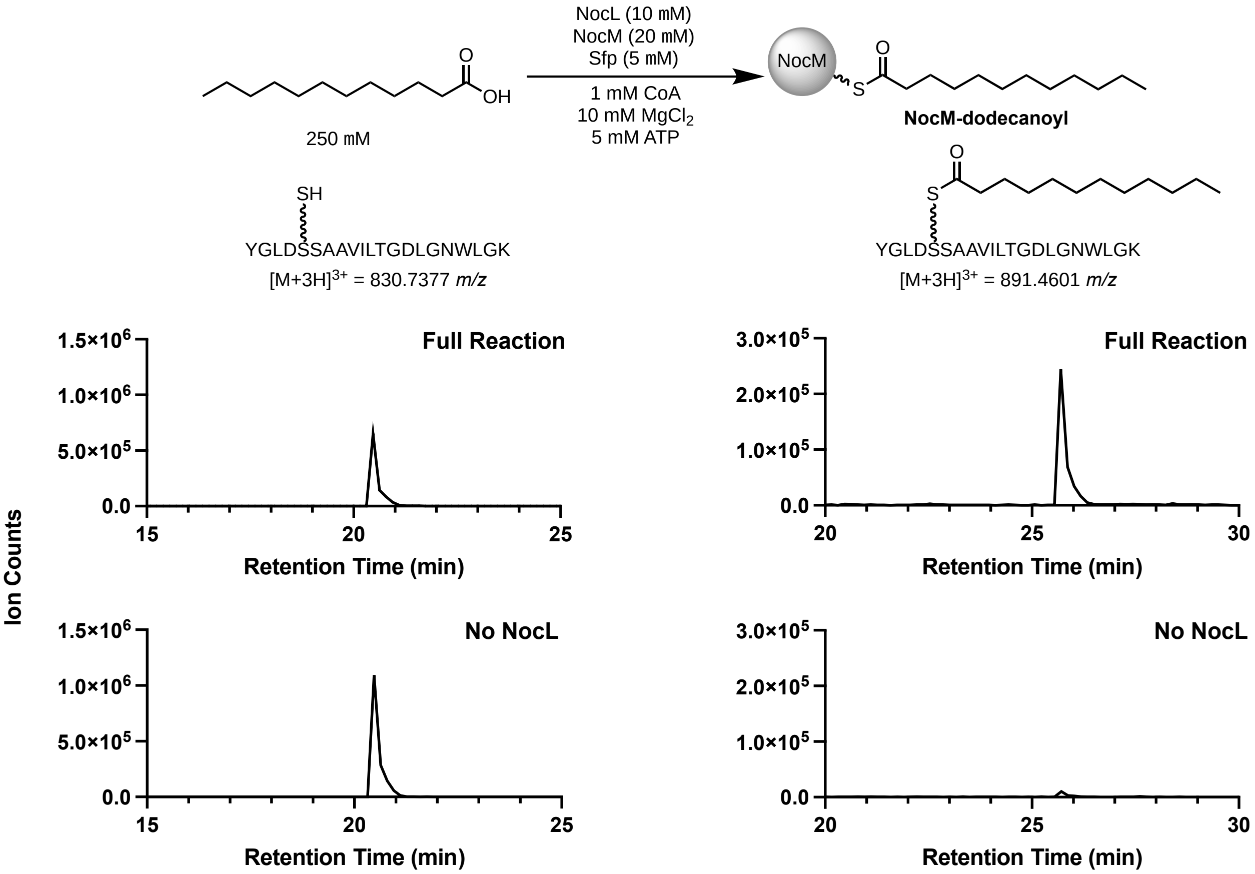

Figure S13. Representative extracted ion chromatograms (EICs) depicting loading of dodecanoic acid onto NocM by NocL. Assays and negative controls were performed in triplicate.

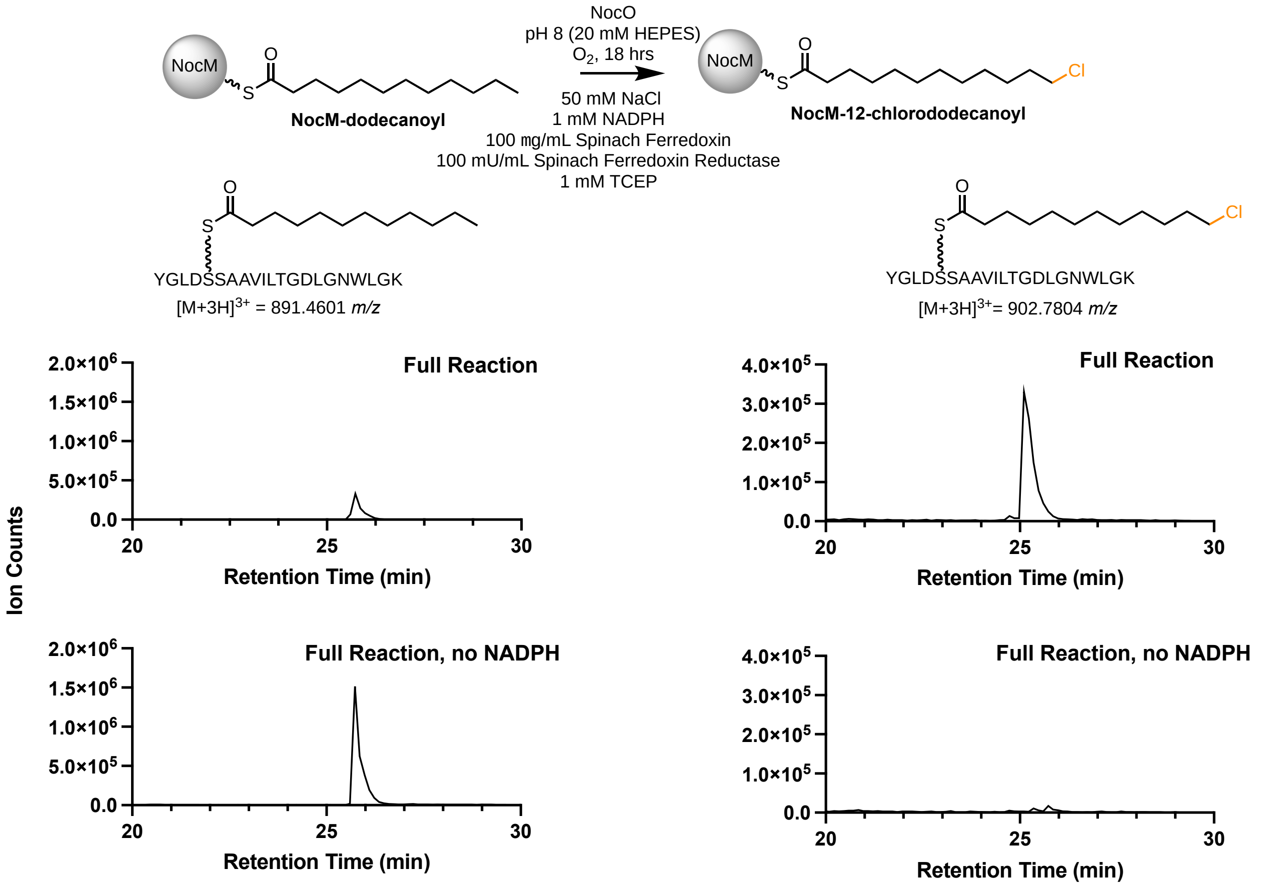

Figure S14. Representative EICs depicting chlorination of NocM-dodecanoyl thioester by purified NocO without a supplemented iron source *in vitro*. Assays and negative controls were performed in triplicate.

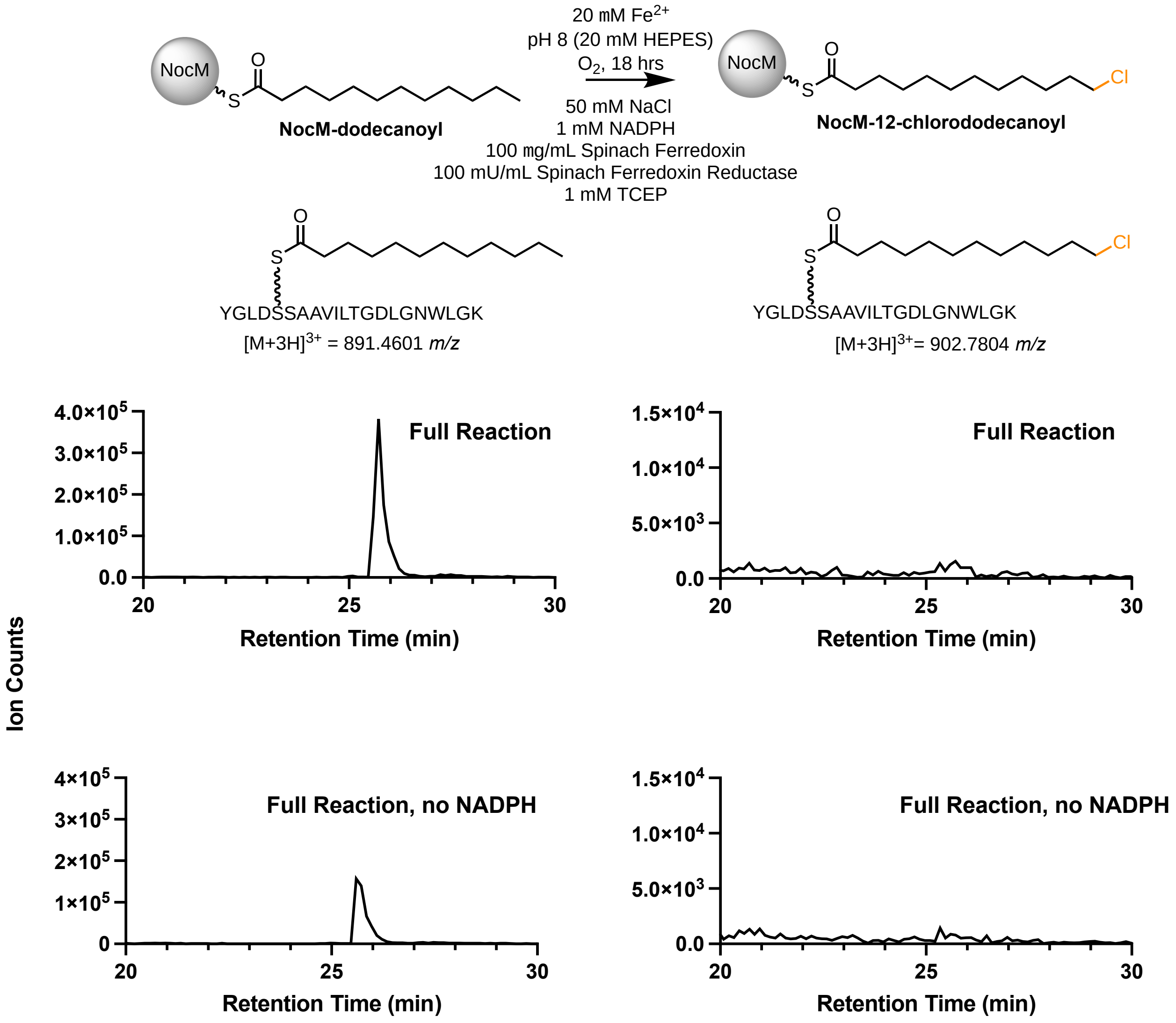

Figure S15. Representative EICs depicting lack of chlorination of NocM-dodecanoyl thioester in the absence of NocO. Assays and negative controls were performed in triplicate.

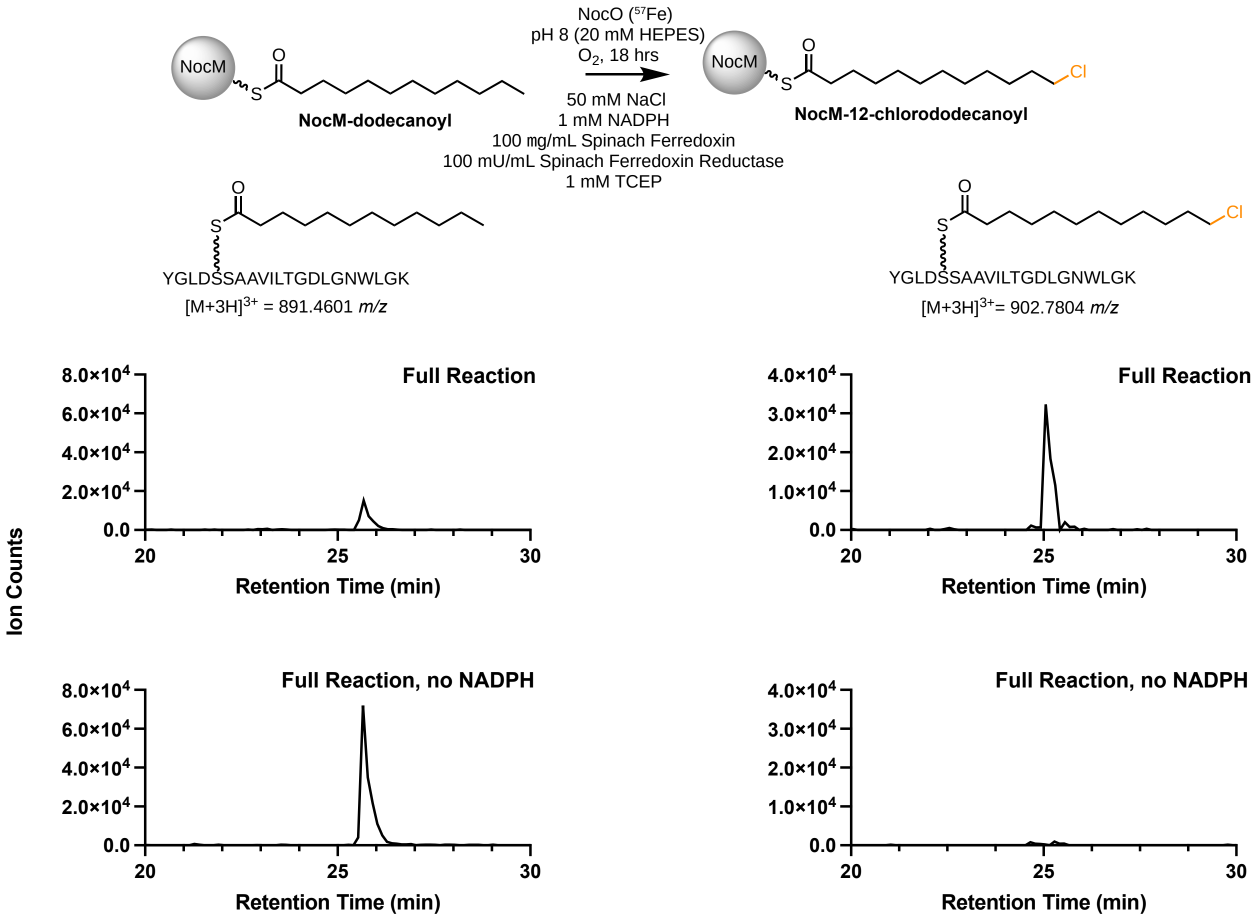

Figure S16. Representative EICs depicting chlorination of NocM-dodecanoyl thioester by as-isolated ^{57}Fe-enriched NocO *in vitro*. Assays and negative controls were performed in triplicate.

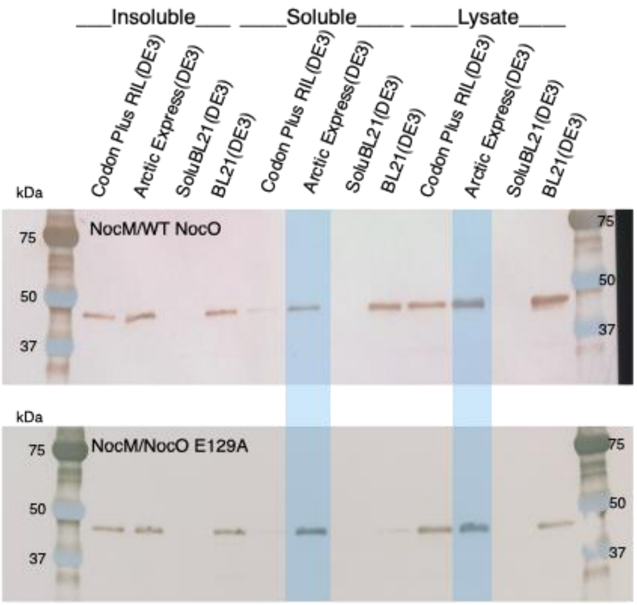

Figure S17. α-His Western blot depicting relative expression levels and solubility of WT NocO and the NocO variant NocO E129A when both are coexpressed with NocM in various BL21(DE3) derivatives. Lysates were prepared and clarified as described in above method and insoluble fractions were prepared via resuspension in 2% SDS. All samples were diluted 5X in water before addition into 2X Laemmli Buffer (Bio-Rad). Samples were run on a 10–20% Wedgewell Tris-Glycine mini precast gel (Invitrogen) alongside Precision Plus All Blue Protein Standards (Bio-Rad). SoluBL21(DE3) was sourced from Gelantis and Codon Plus RIL(DE3) was sourced from Agilent.

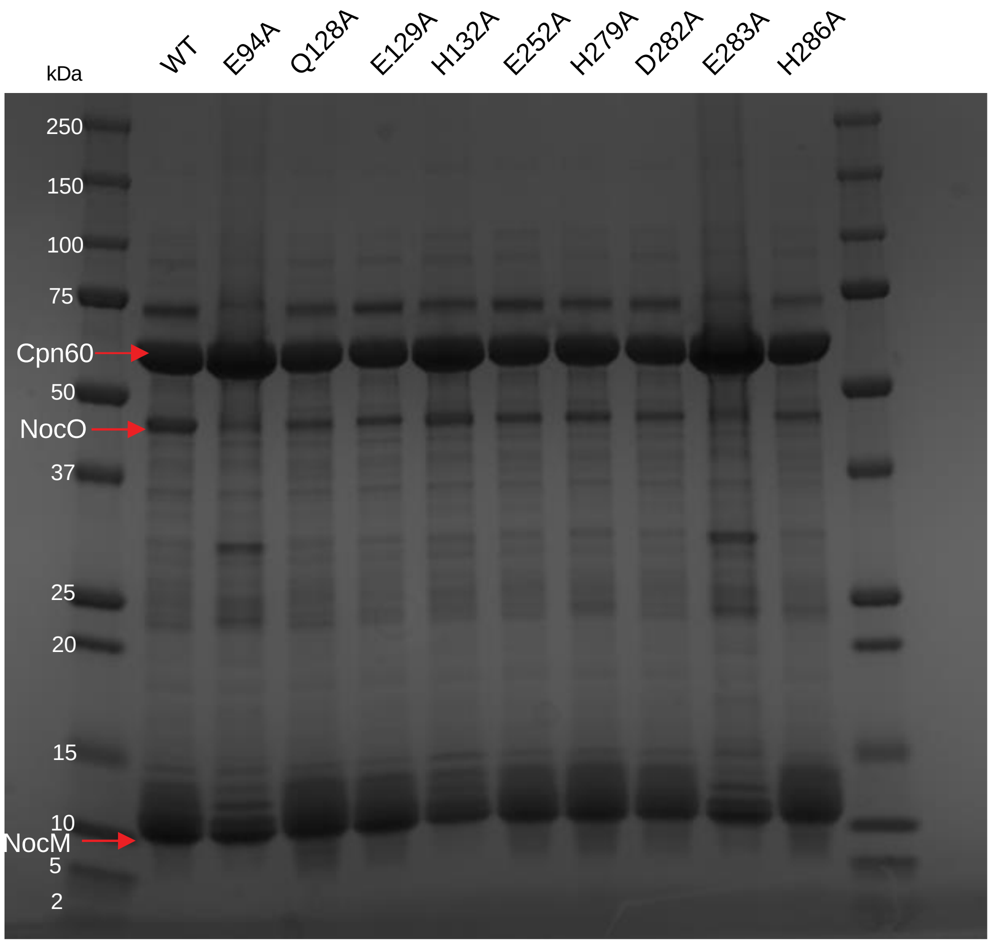

Figure S18. SDS-PAGE depicting NocM/NocO and respective variants as purified from ArcticExpress(DE3). Samples were run on a 10–20% Wedgewell Tris-Glycine mini precast gel alongside Precision Plus Dual Xtra Prestained Protein Standards (Bio-Rad).

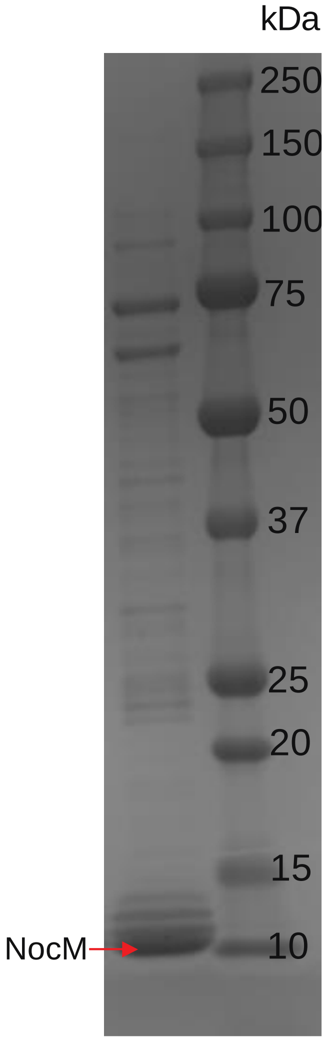

Figure S19. SDS-PAGE depicting purified NocM/empty pET-Duet from ArcticExpress(DE3). Samples were run on a 10–20% Wedgewell Tris-Glycine mini precast gel alongside Precision Plus All Blue Protein Standards (Bio-Rad).

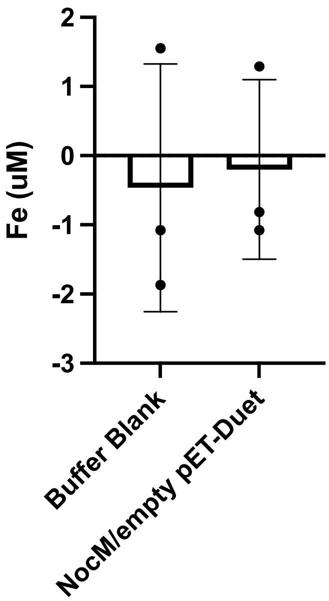

Figure S20. Iron content as determined by Ferene-S assay in a 10 µM sample of NocM and a buffer blank. Bars represent average of triplicate measurements (mean $\pm$ SD).

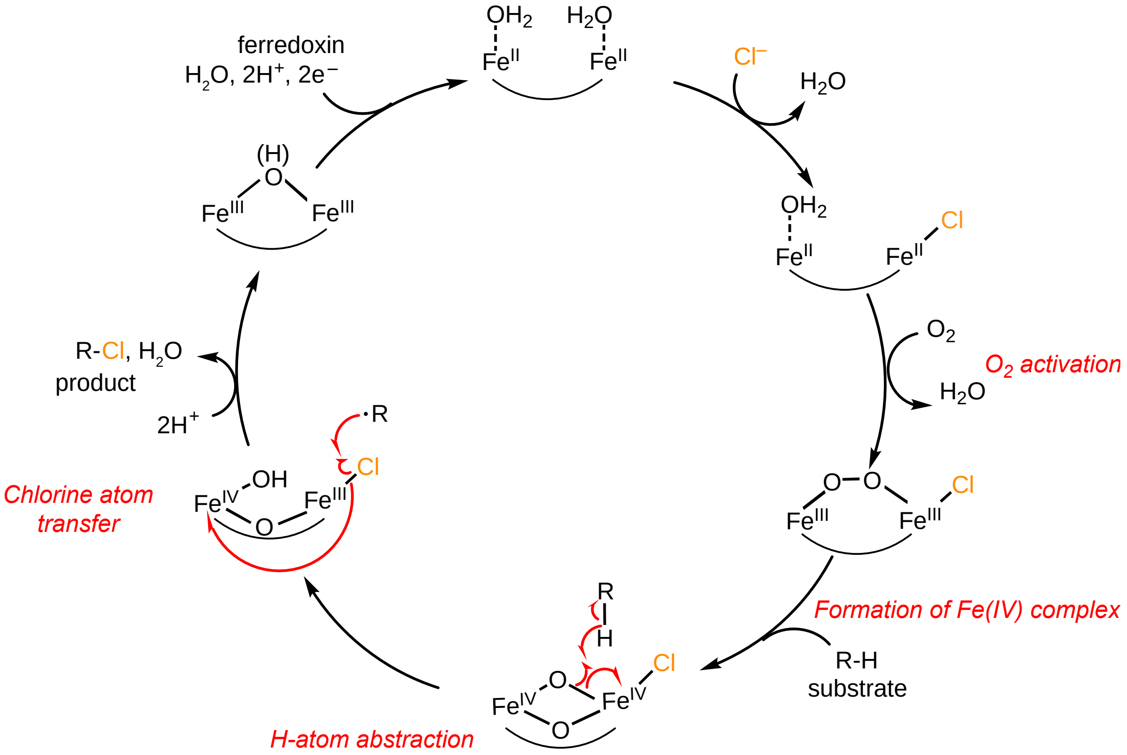

Figure S21. Mechanistic hypothesis for chlorination by diiron halogenases.

References:

(1) Kennedy, M. C.; Kent, T. A.; Emptage, M.; Merkle, H.; Beinert, H.; Münck, E. Evidence for the Formation of a Linear [3Fe-4S] Cluster in Partially Unfolded Aconitase. *J. Biol. Chem.* **1984**, *259* (23), 14463–14471. https://doi.org/10.1016/S0021-9258(17)42622-6.

(2) Nakamura, H.; Schultz, E. E.; Balskus, E. P. A New Strategy for Aromatic Ring Alkylation in Cylindrocyclophane Biosynthesis. *Nat. Chem. Biol.* **2017**, *13* (8), 916–921. https://doi.org/10.1038/nchembio.2421.
